# Supplementary material for: Automatic Detection of Microsleep Episodes With Deep Learning
Source: Front Neurosci. 2021 Mar 24;15:564098. doi: 10.3389/fnins.2021.564098 (PMC8024556; doi:10.3389/fnins.2021.564098)
Supplement: Supplementary file 1 [file Table_1.DOCX]

# Supplementary Figures

**Supplementary Figure S1.** Expert (top) and automatic soring with one algorithm (CNN with 16-s window; bottom) of the 12 patients in the validation set. Scoring was performed with the resolution of one sample; for the illustration, we coarsened the result to a resolution of 0.5 s (100 samples), i.e. the most frequent class within an interval was plotted. The MWT lasted 40 min and was supposed to be terminated earlier if three consecutive 30-s epochs of N1 or one epoch of any other sleep stage occurred. The time axis is in minutes till the termination of the MWT. W: wakefulness; MSE: microsleep episodes; ED: episodes of drowsiness; MSEc: microsleep episode candidates. The patient ID is provided at the top of the plot. Patients of the test set are illustrated in Supplementary Figure S2.

| 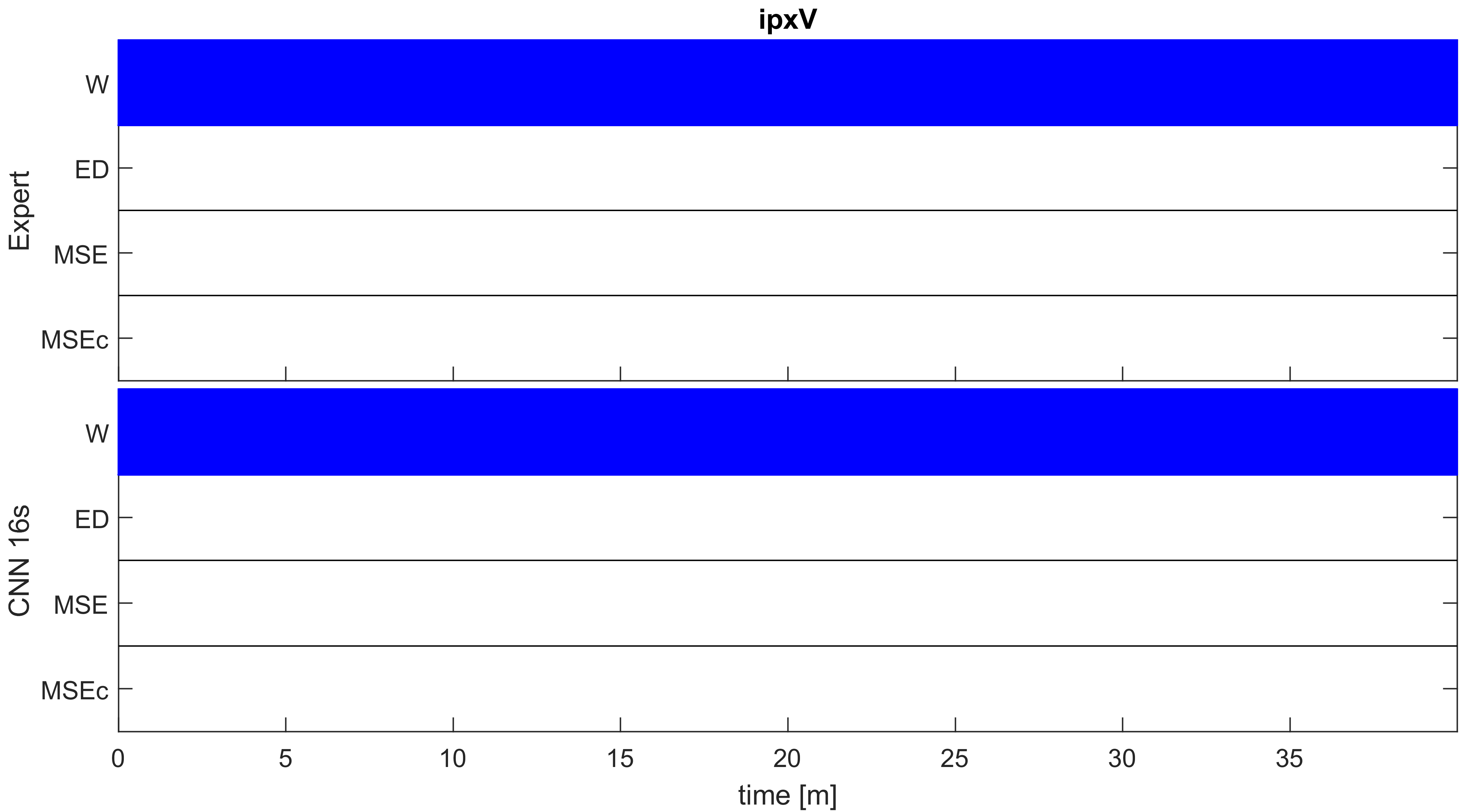 | 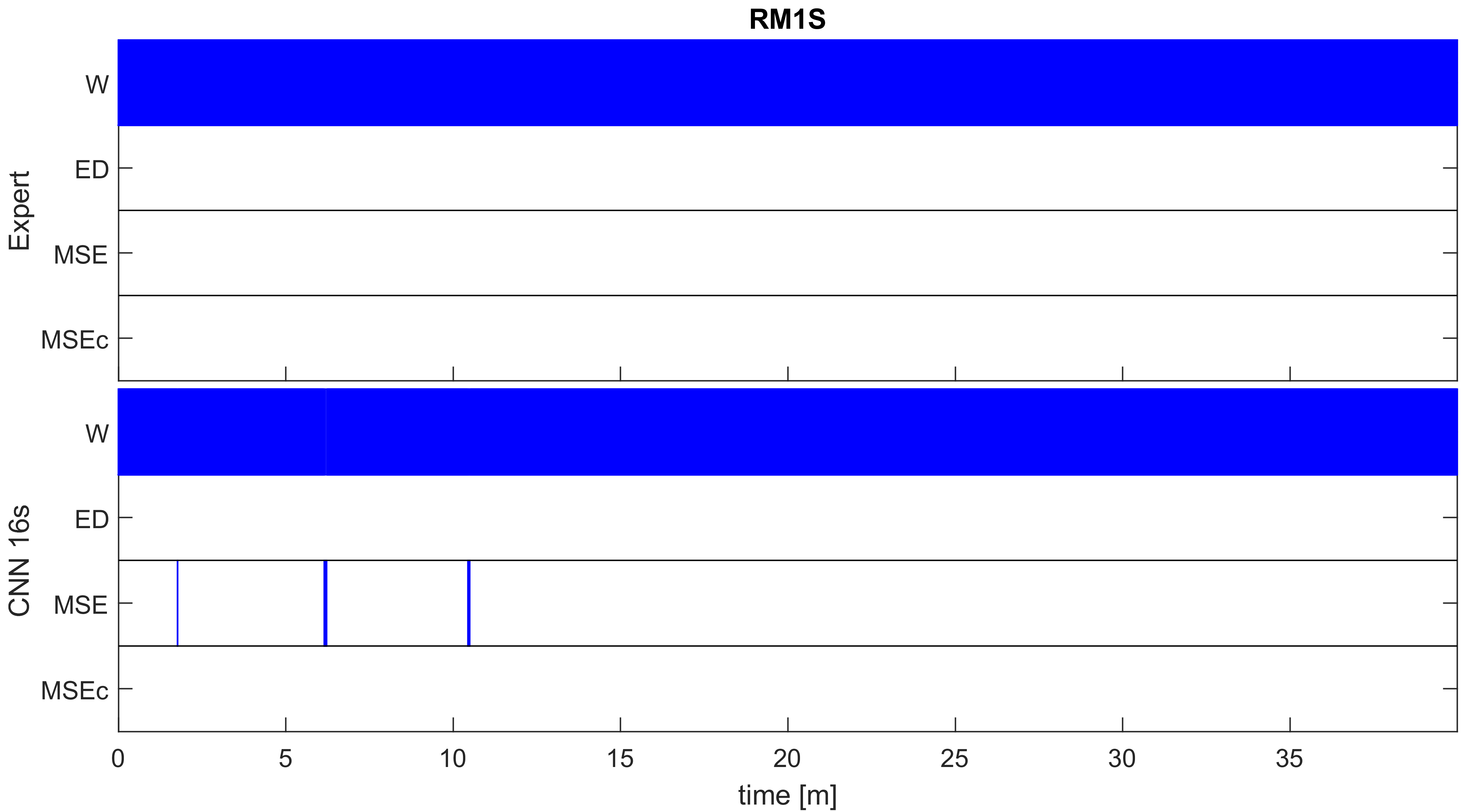 |
| --- | --- |
| 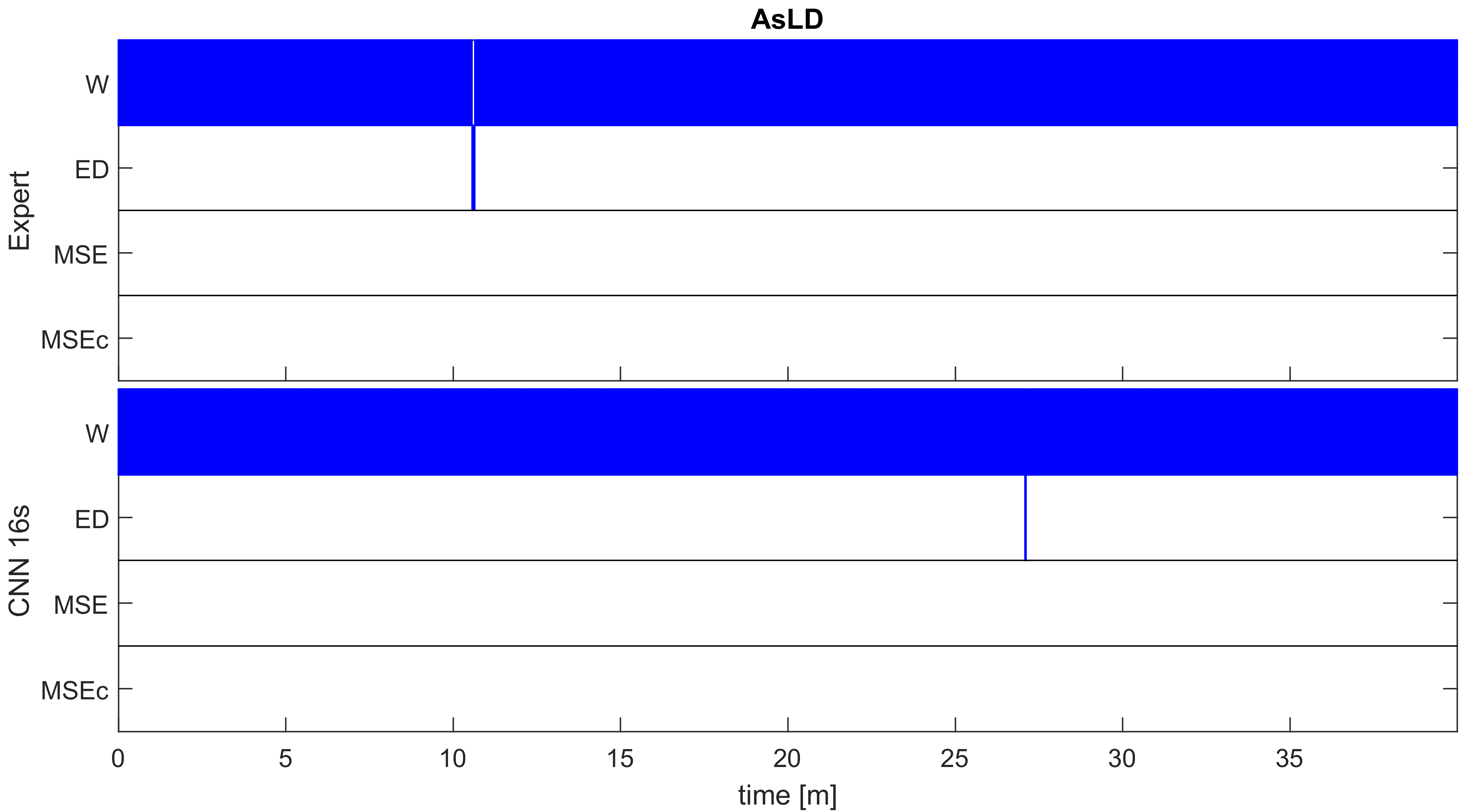 | 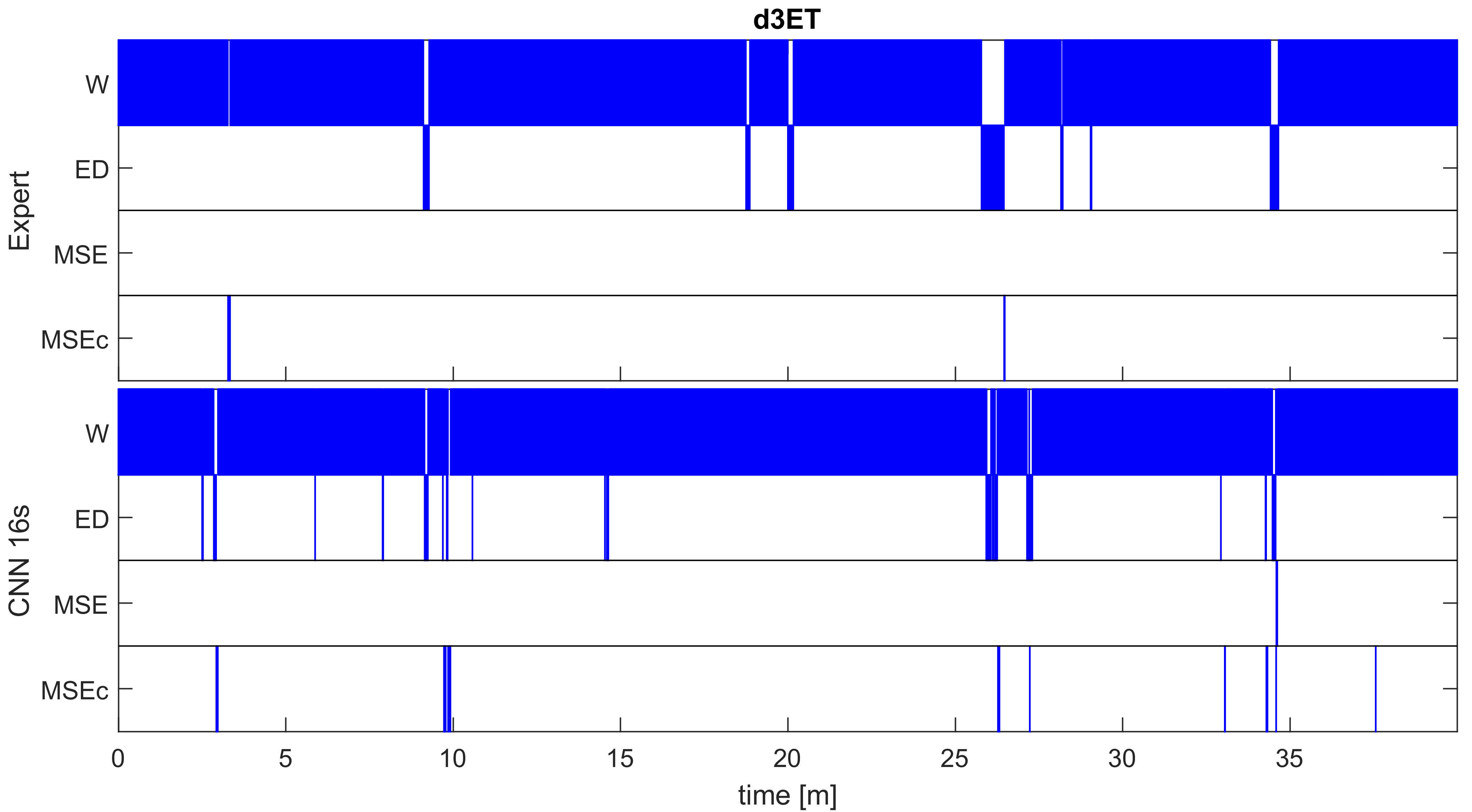 |
| 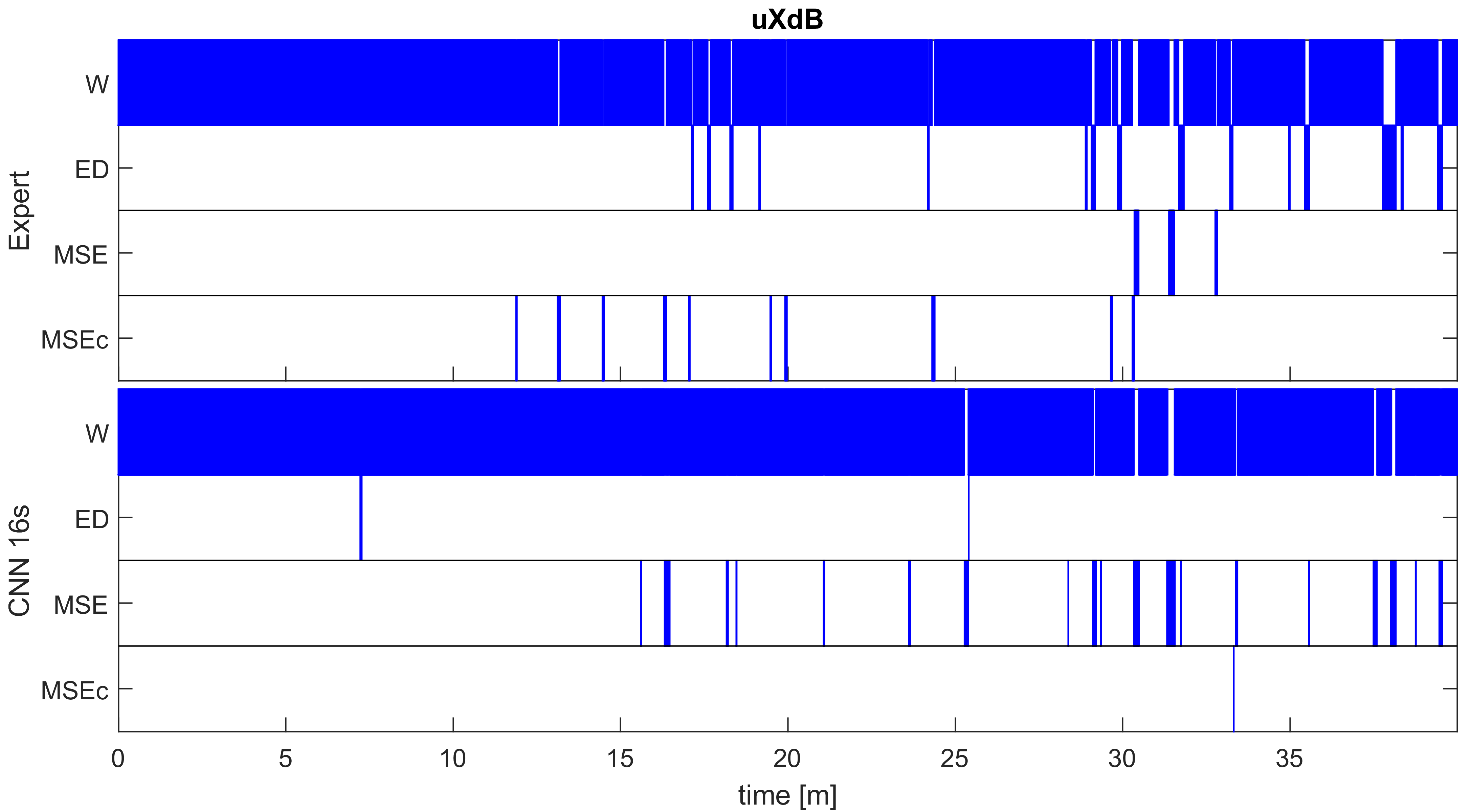 | 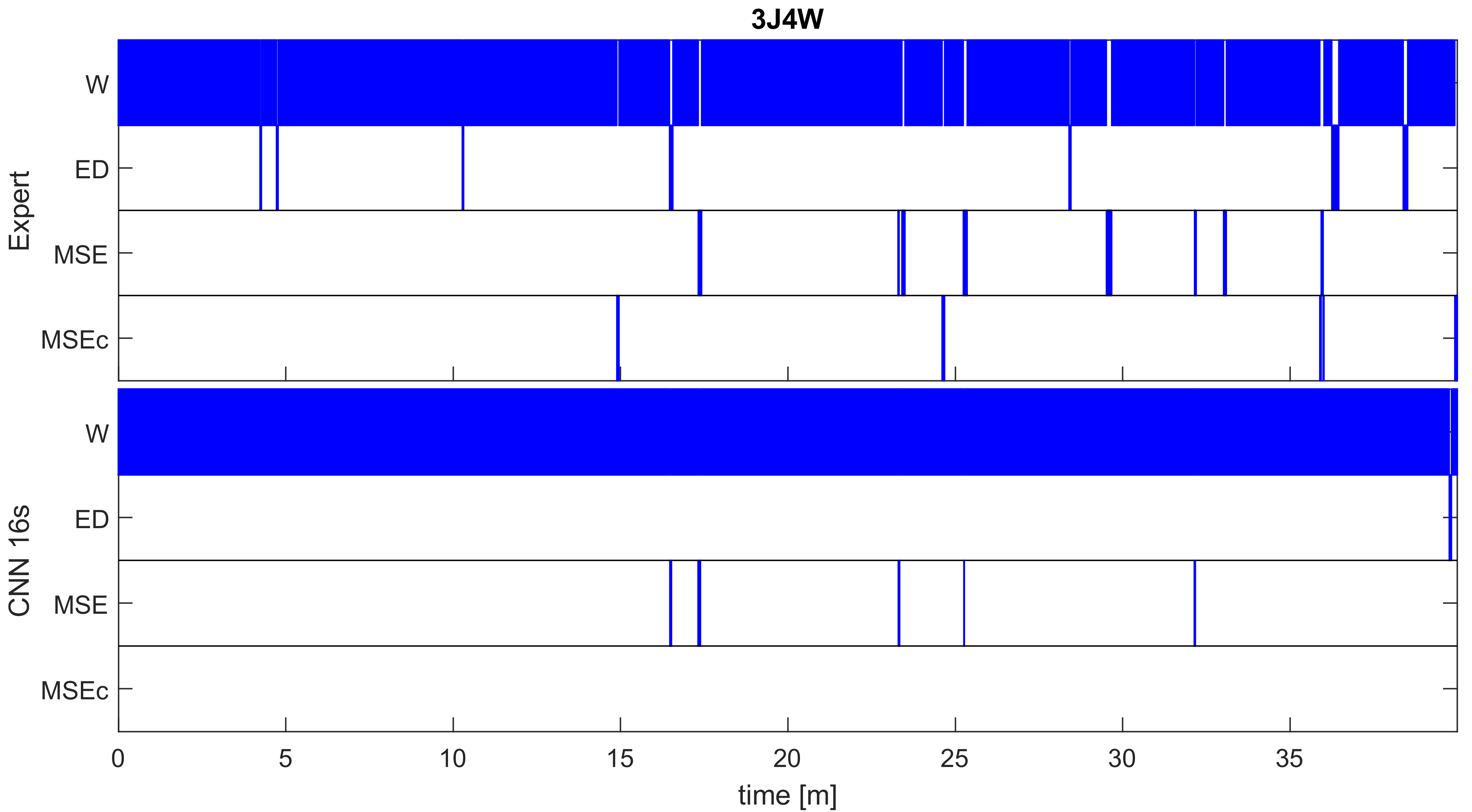 |
| 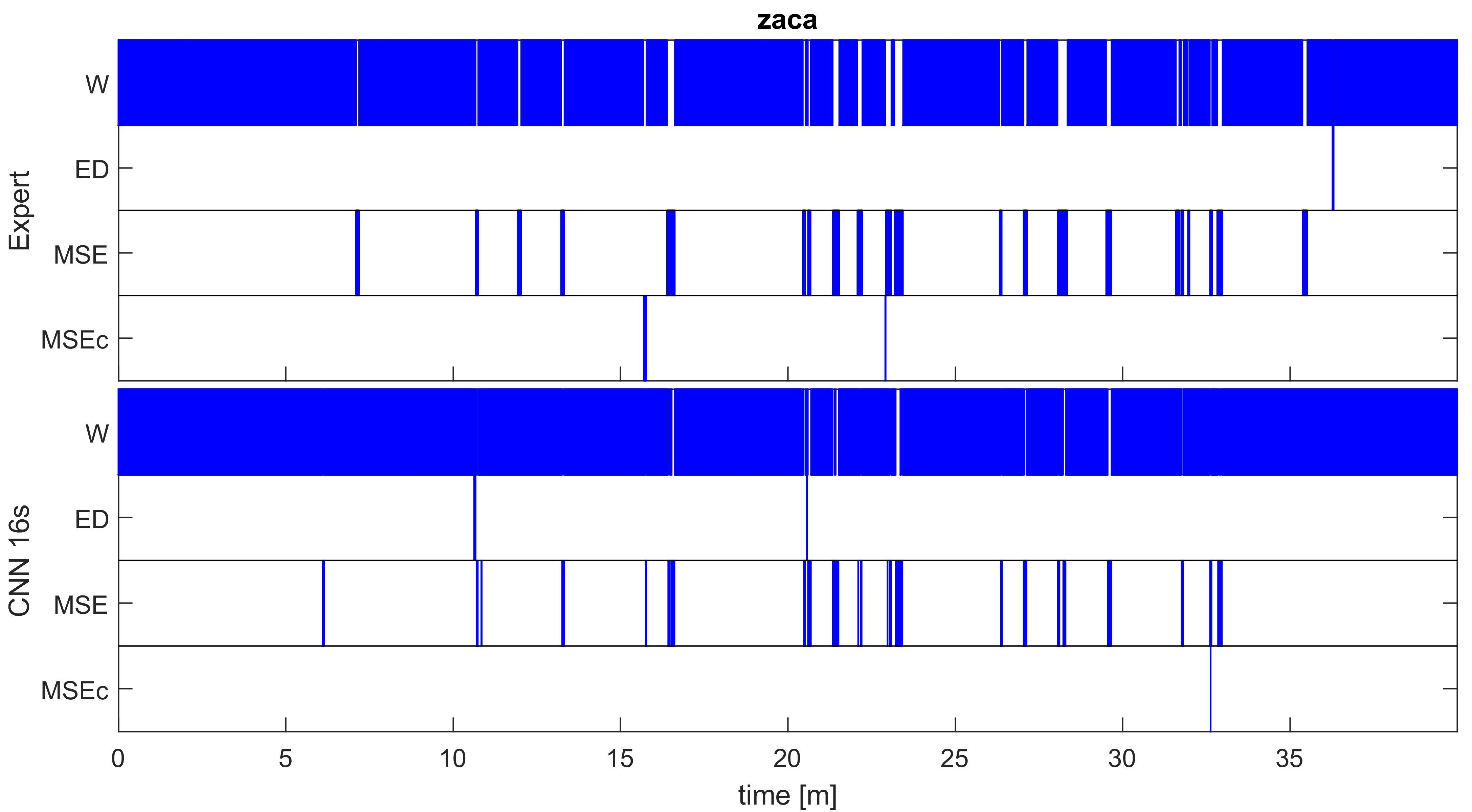 | 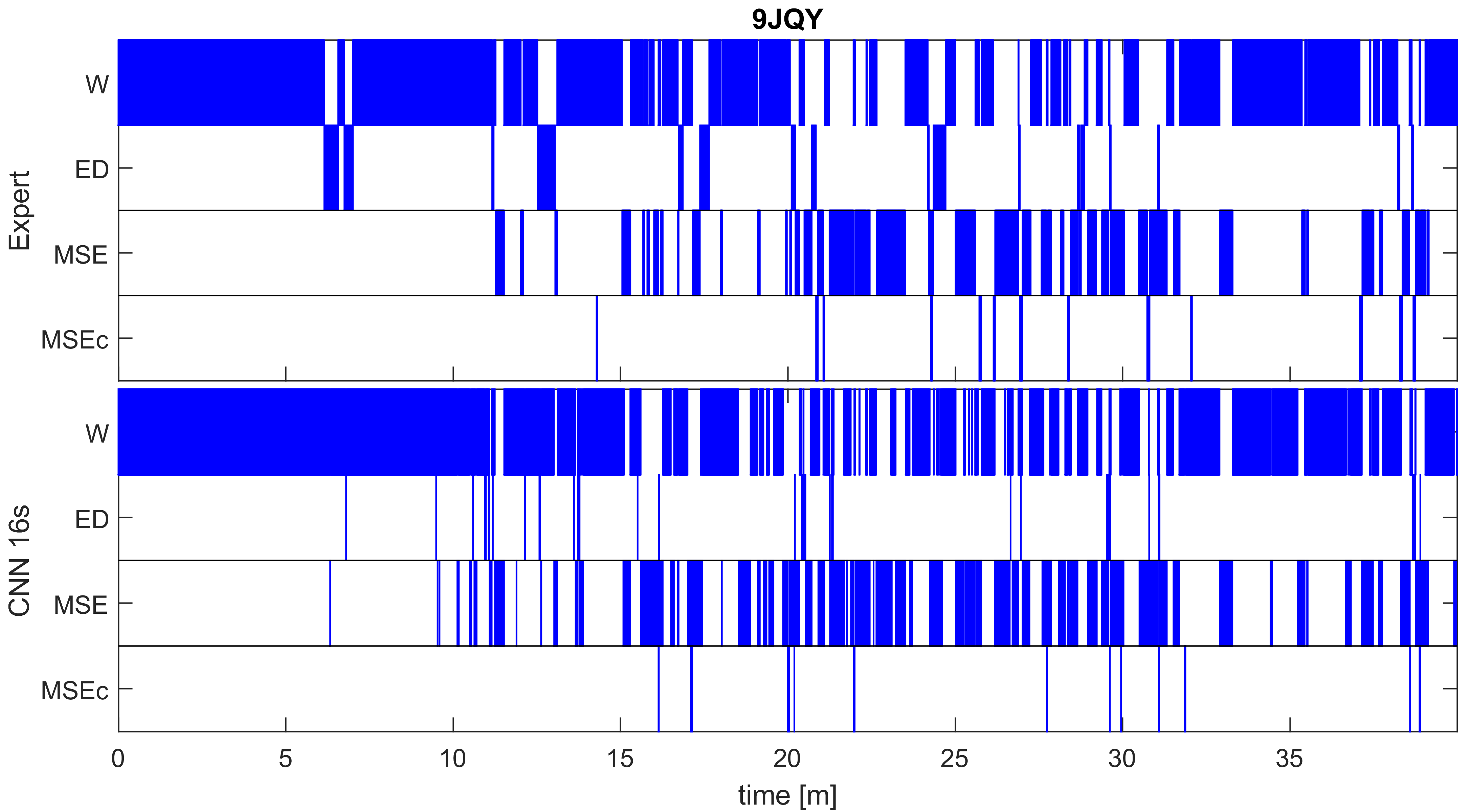 |
| 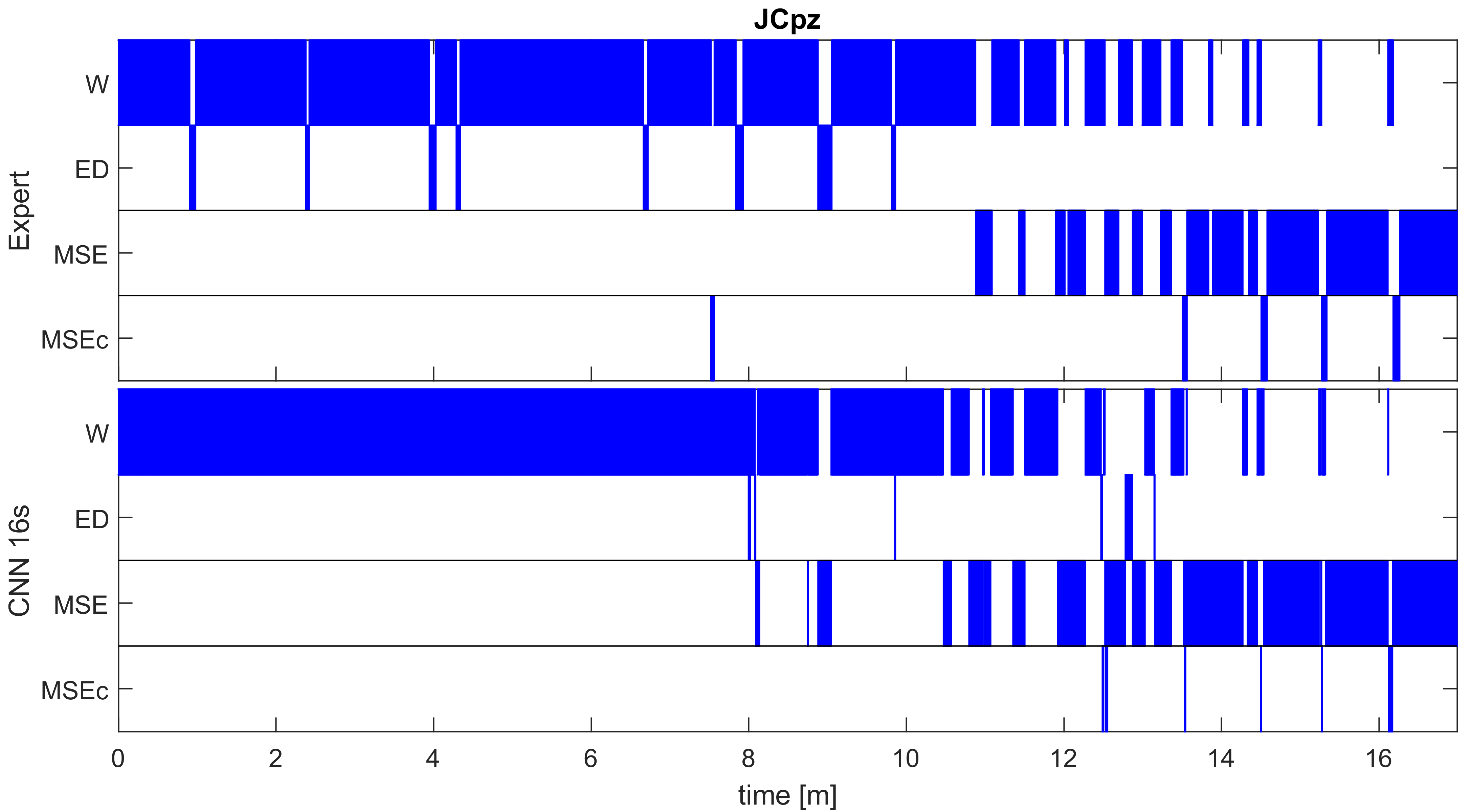 | 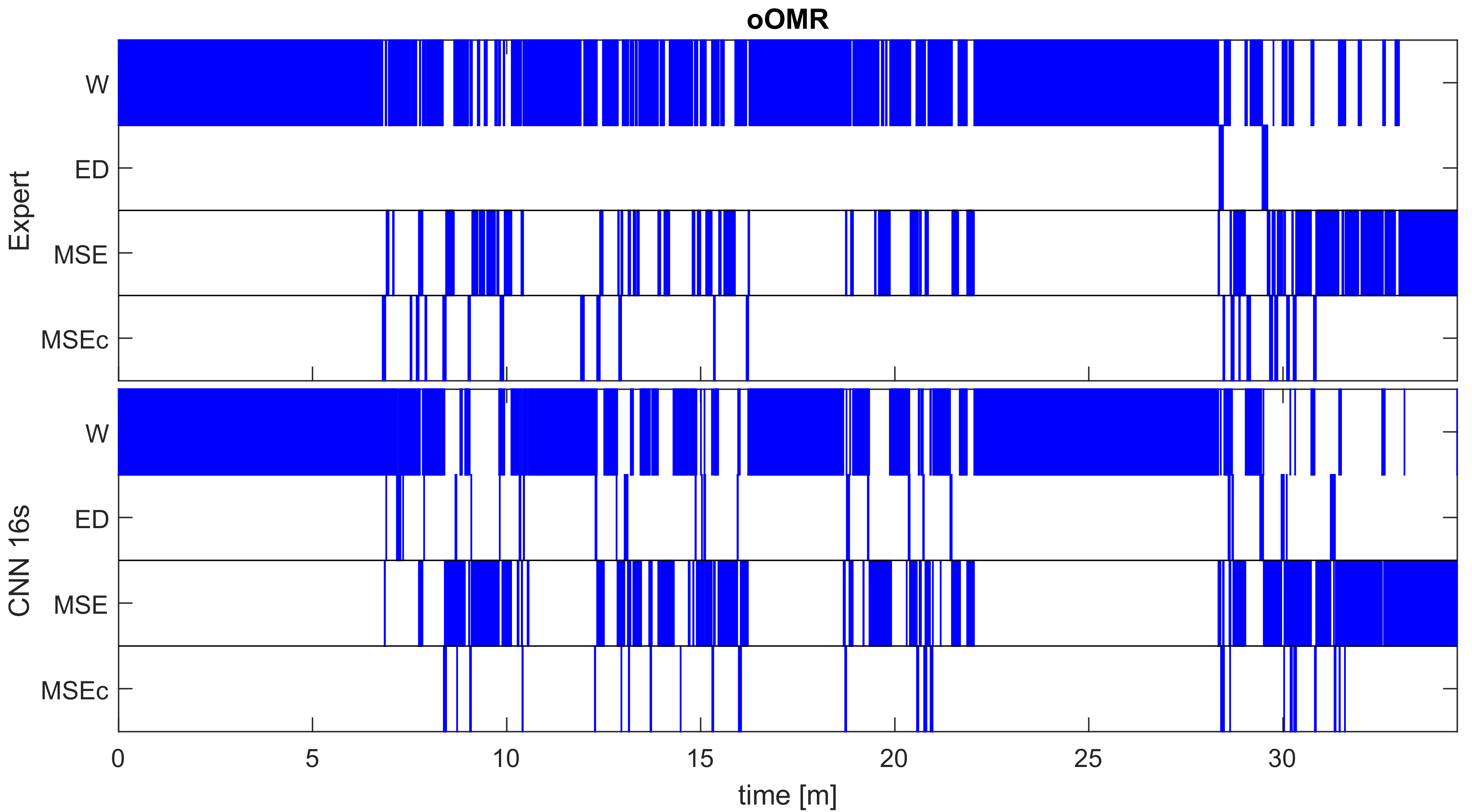 |
| 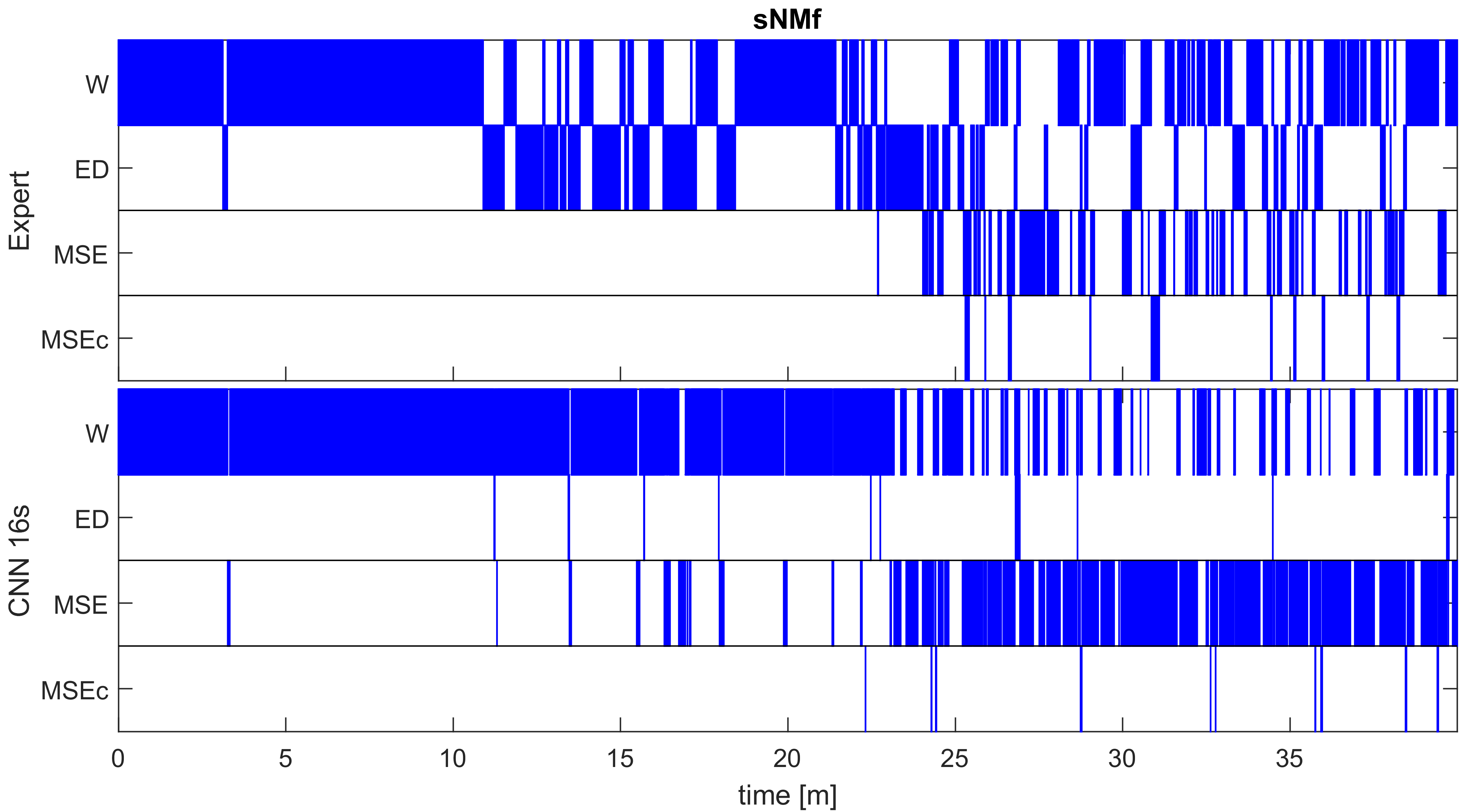 | 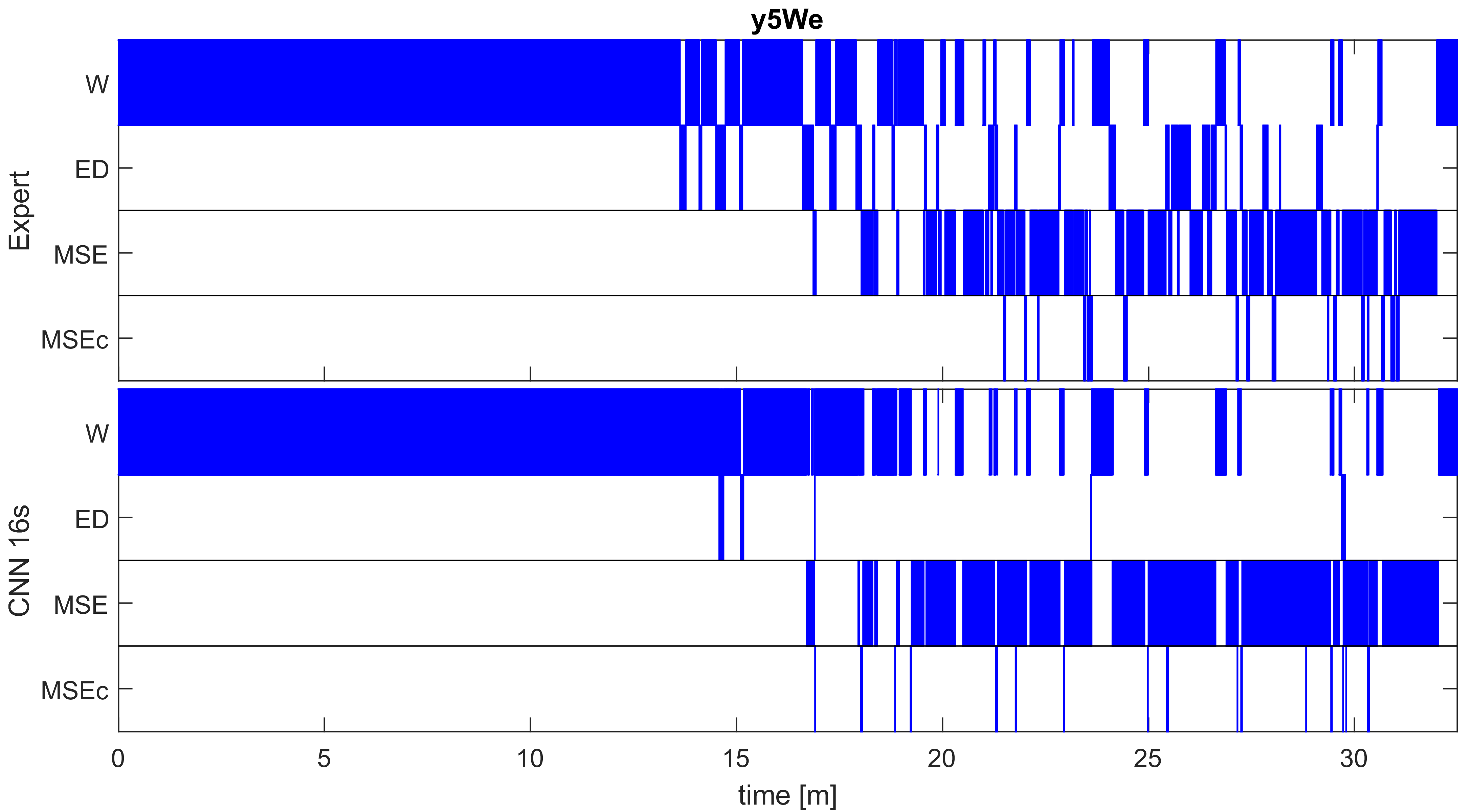 |

**Supplementary Figure S2.** Expert (top) and automatic soring with one algorithm (CNN with 16-s window; bottom) of the 11 patients in the test set. For details see Supplementary Figure S1.

| 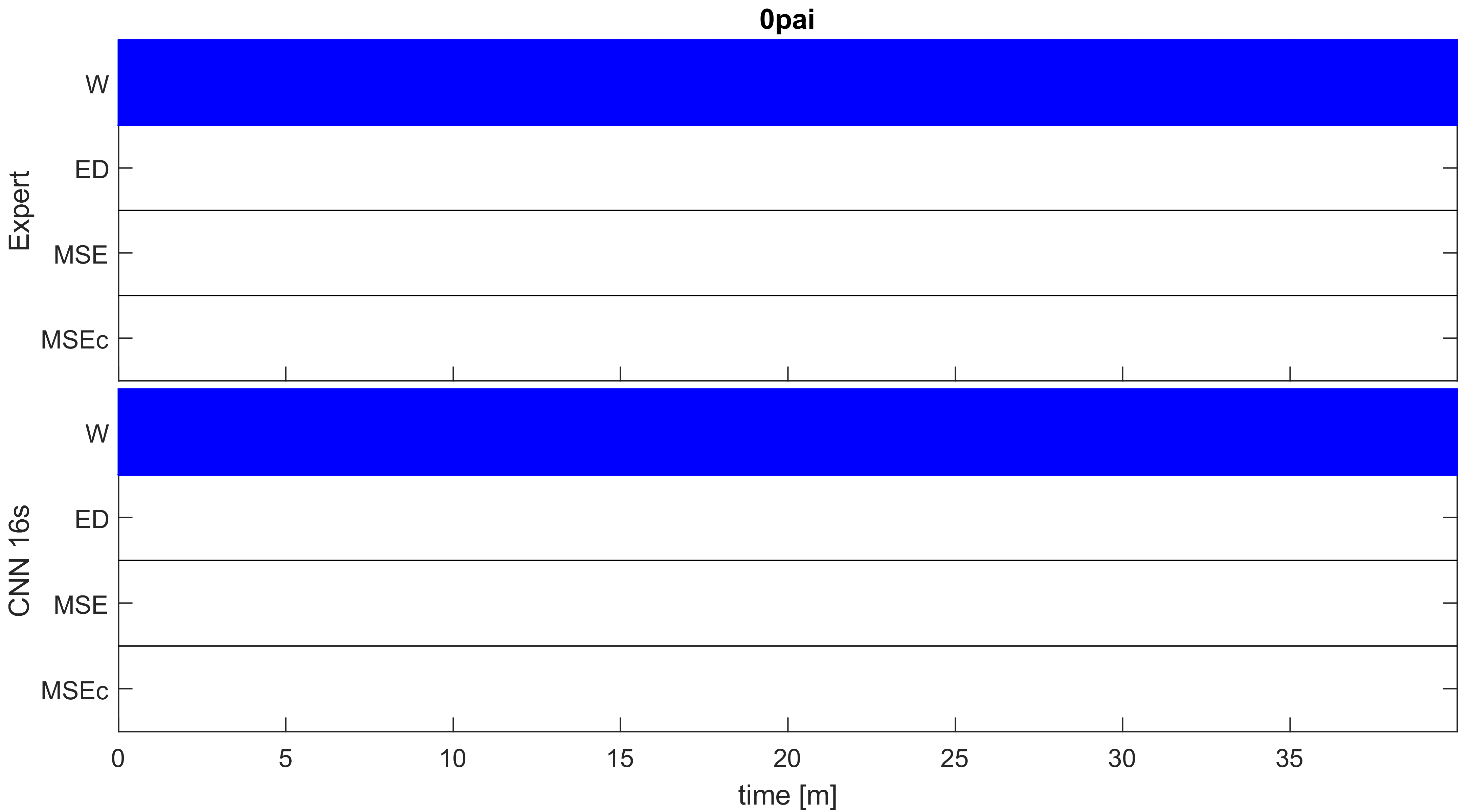 | 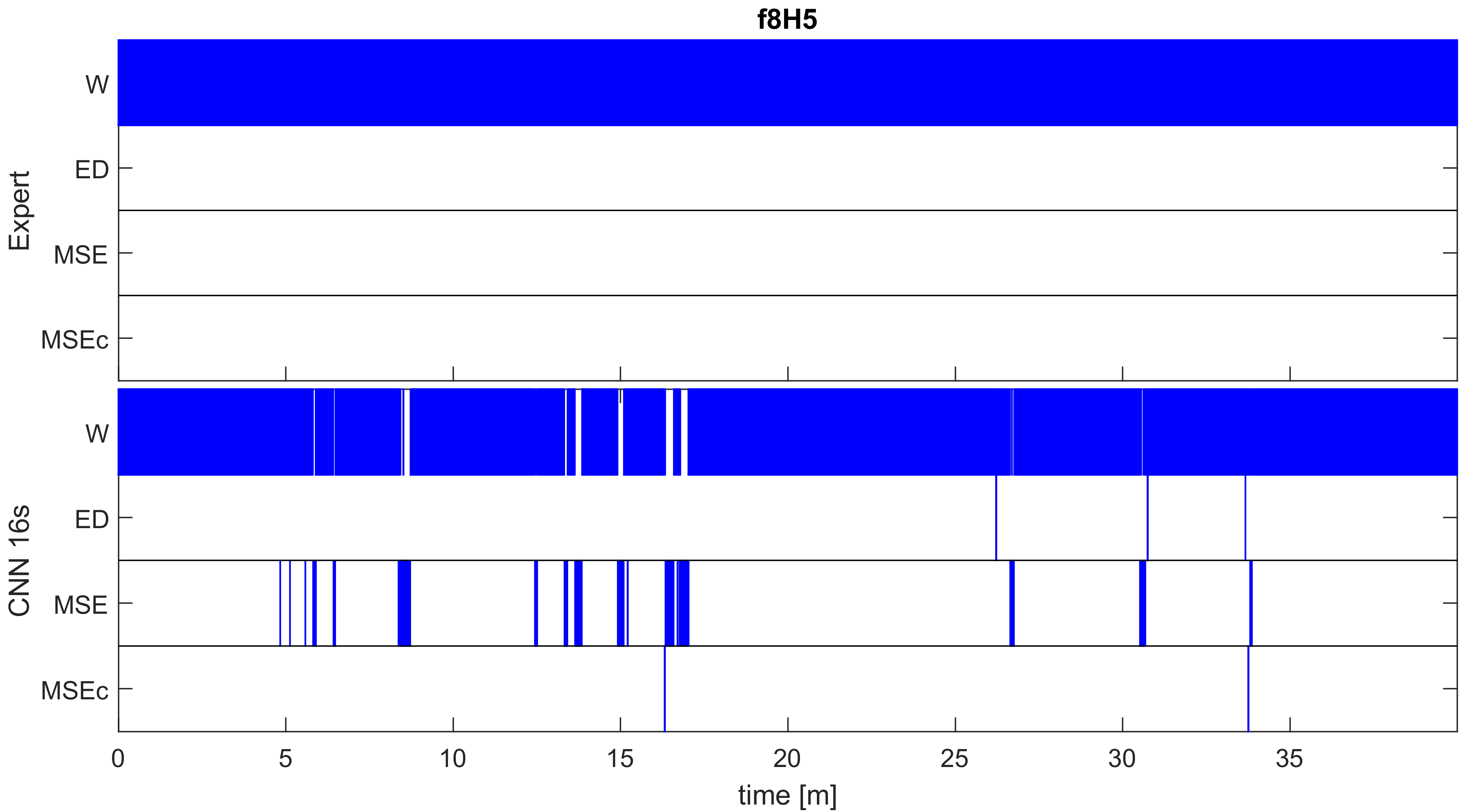 |
| --- | --- |
| 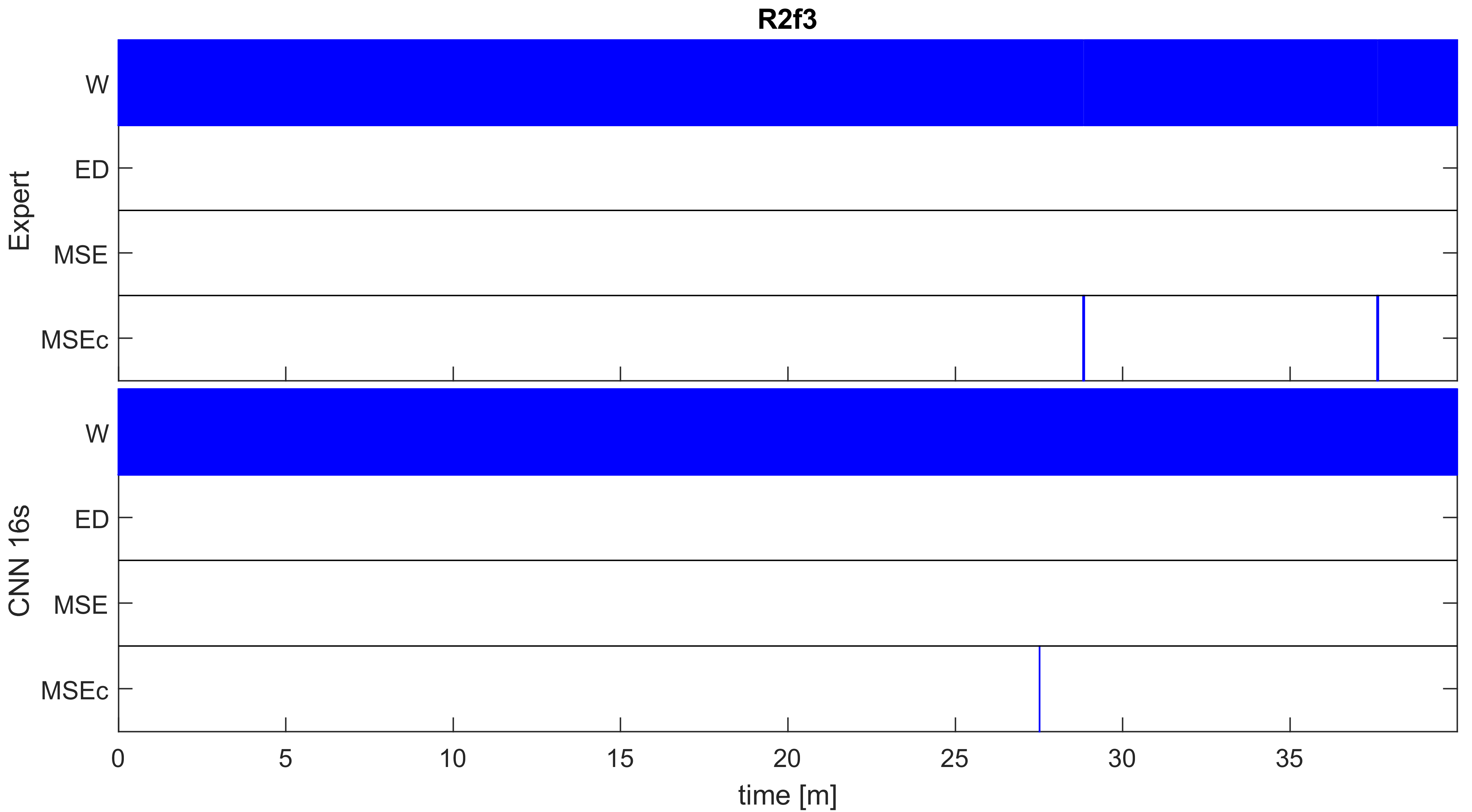 | 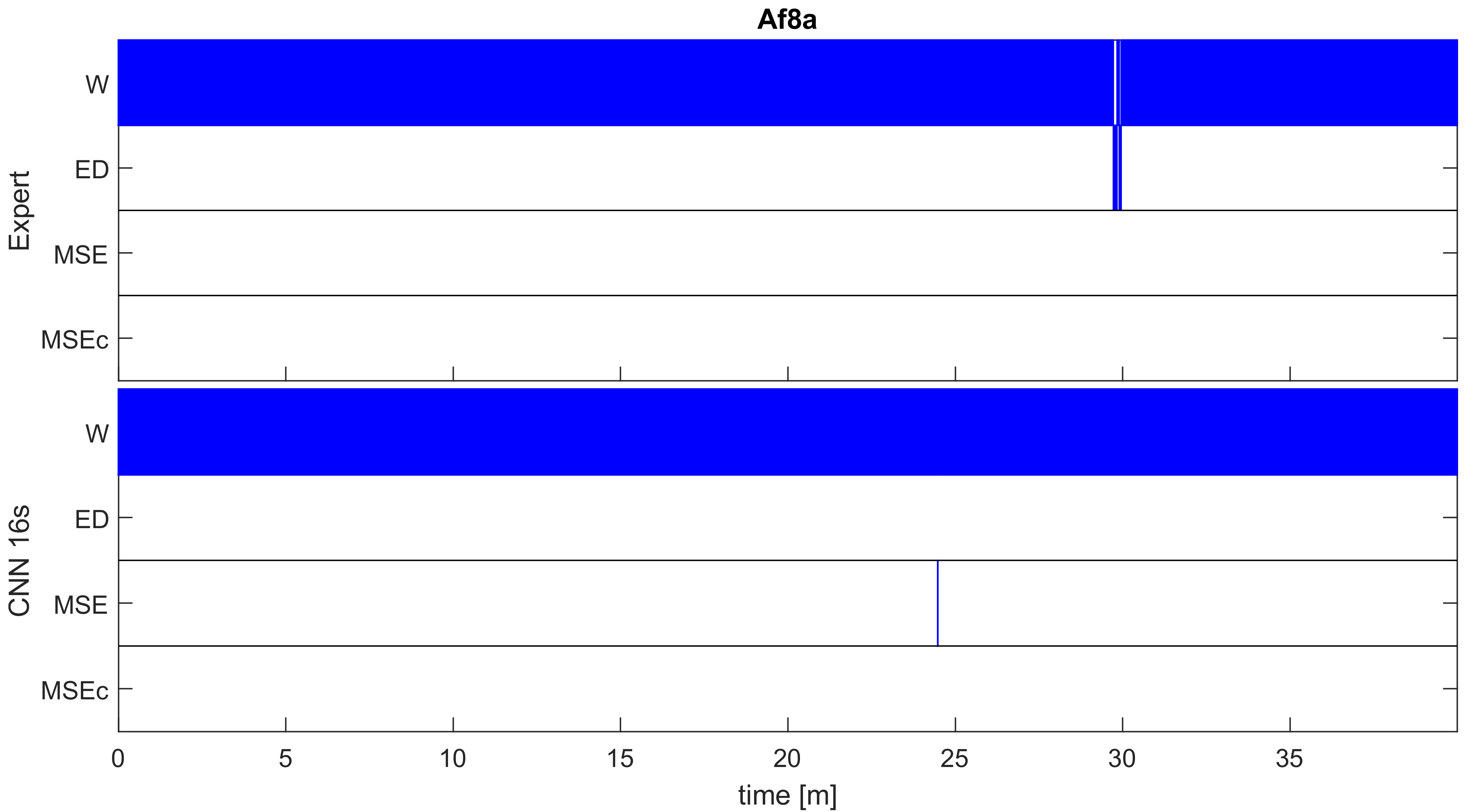 |
| 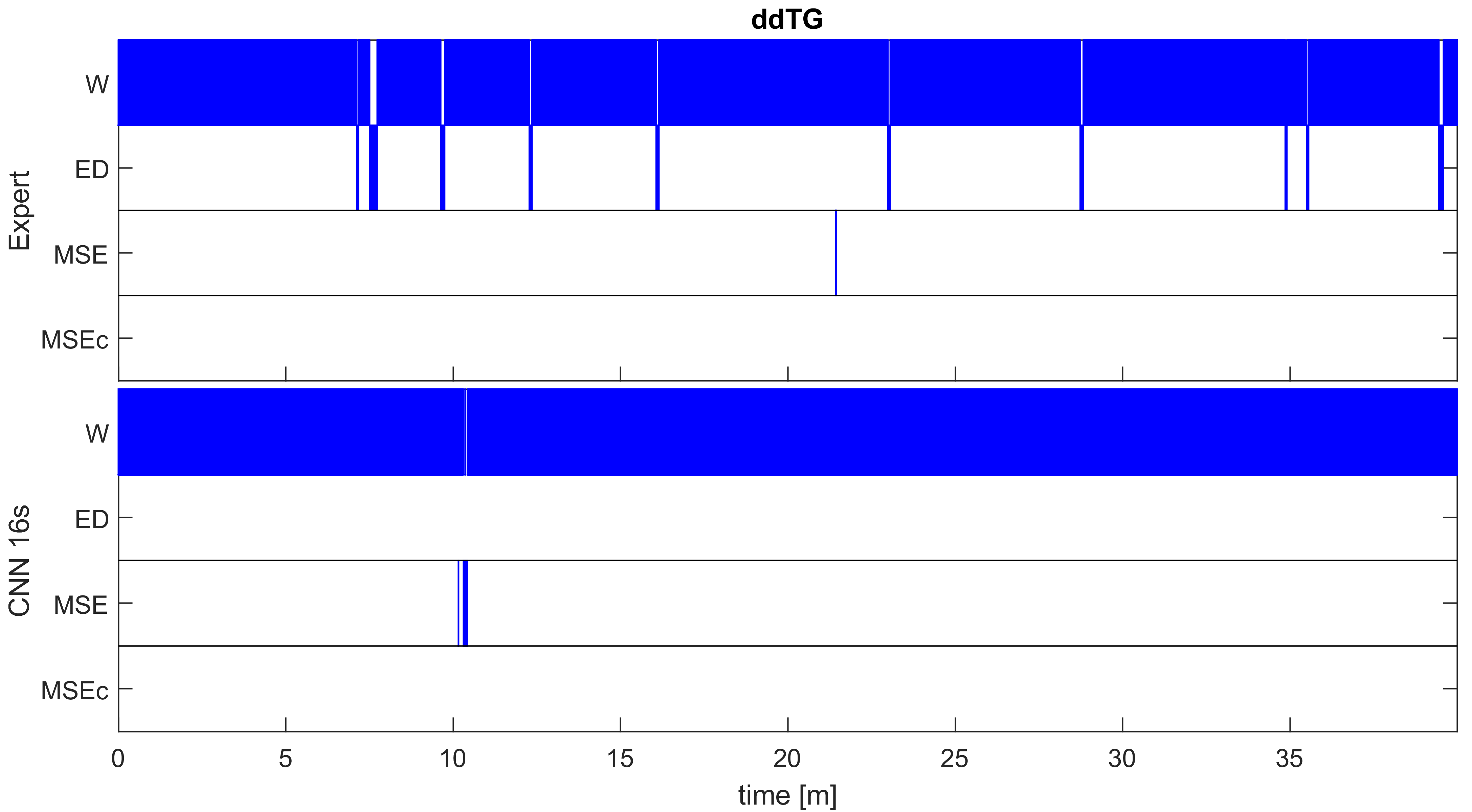 | 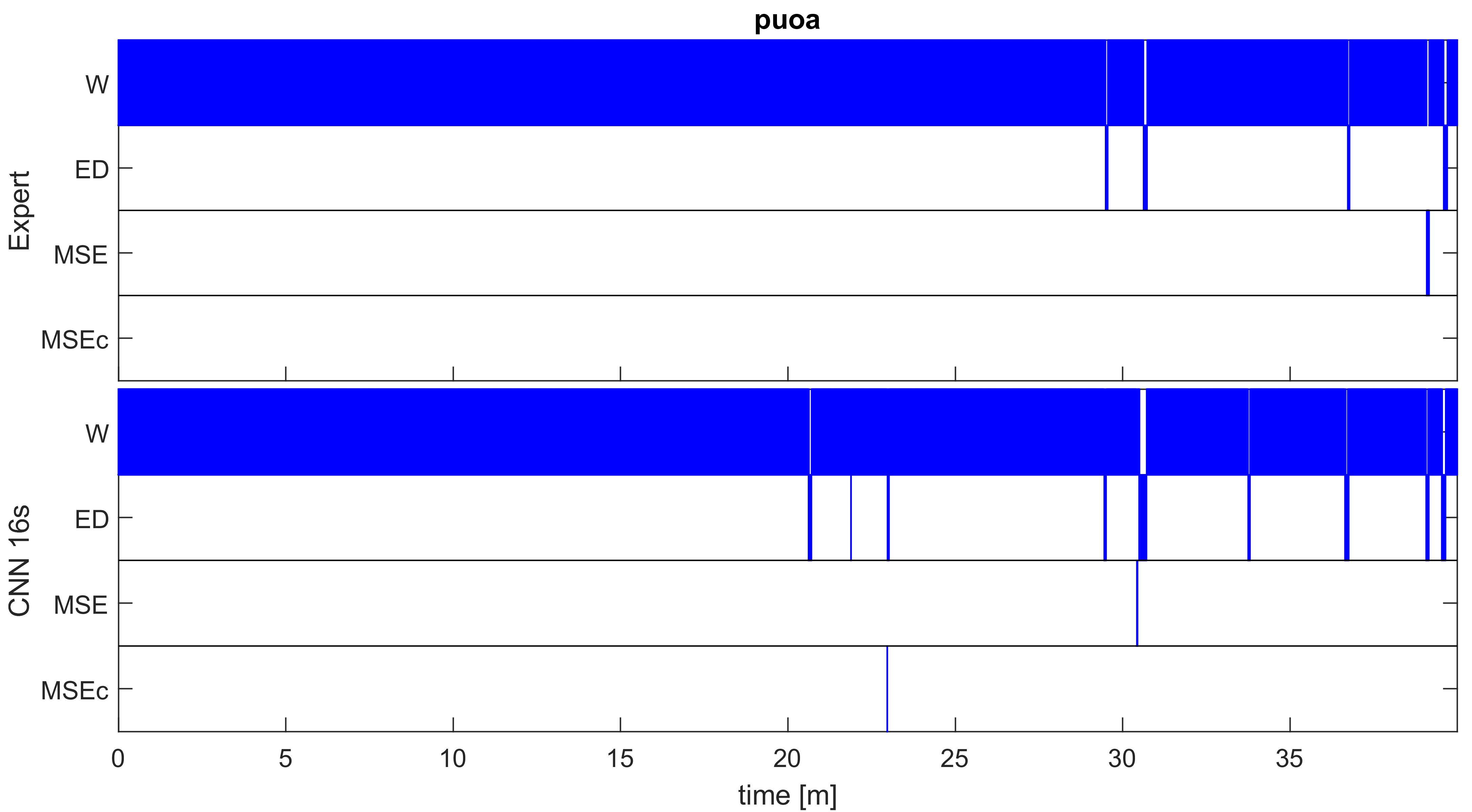 |
| 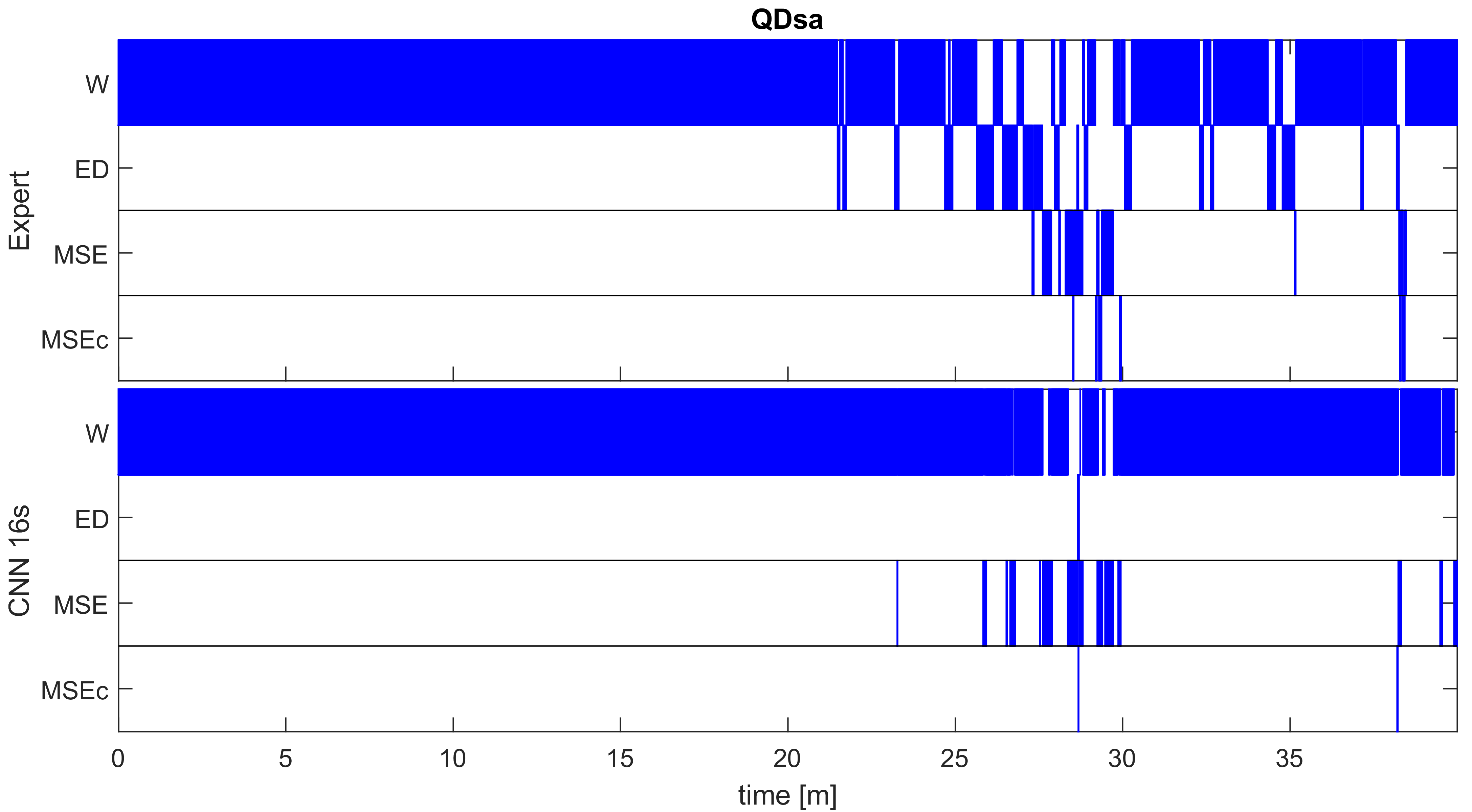 | 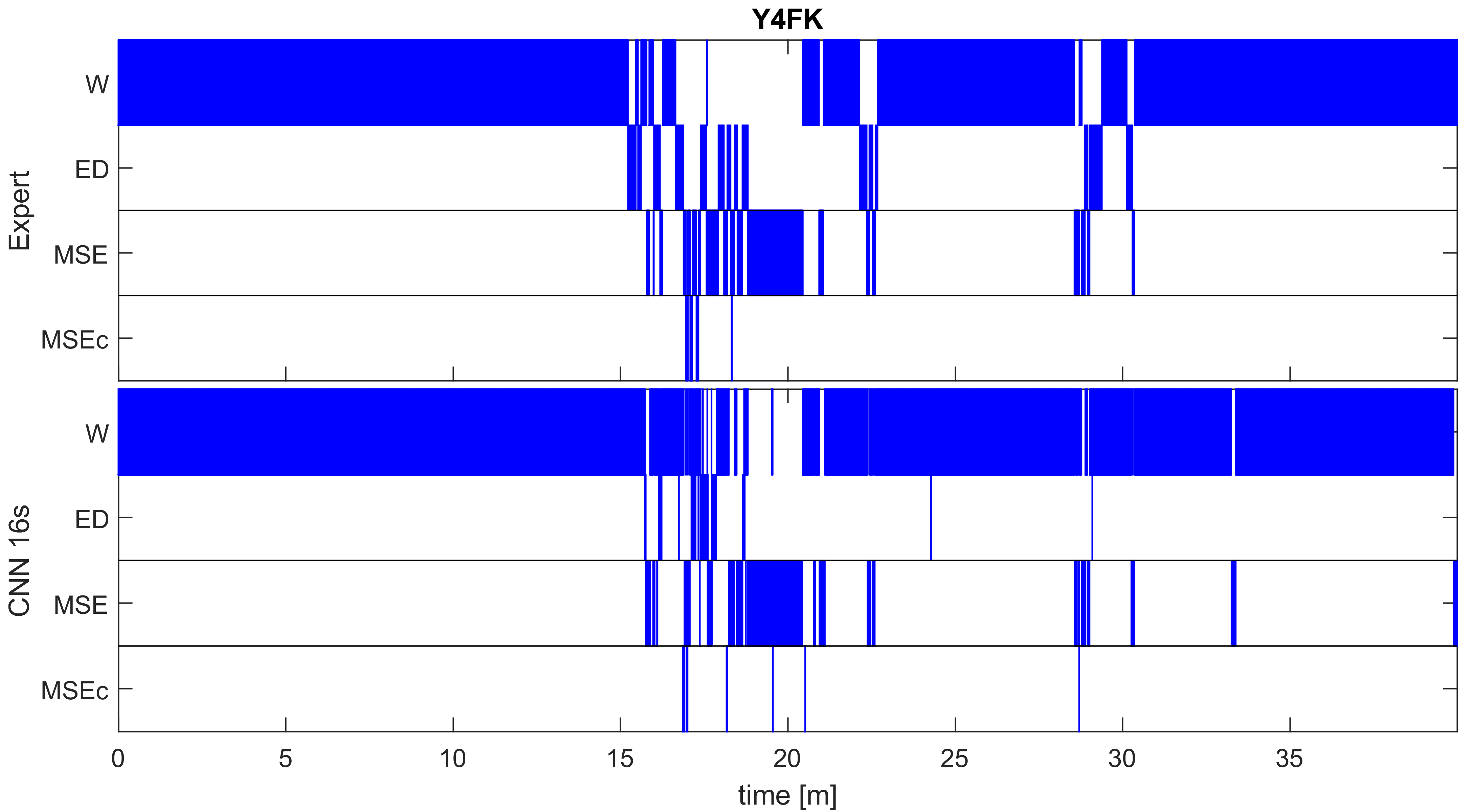 |
| 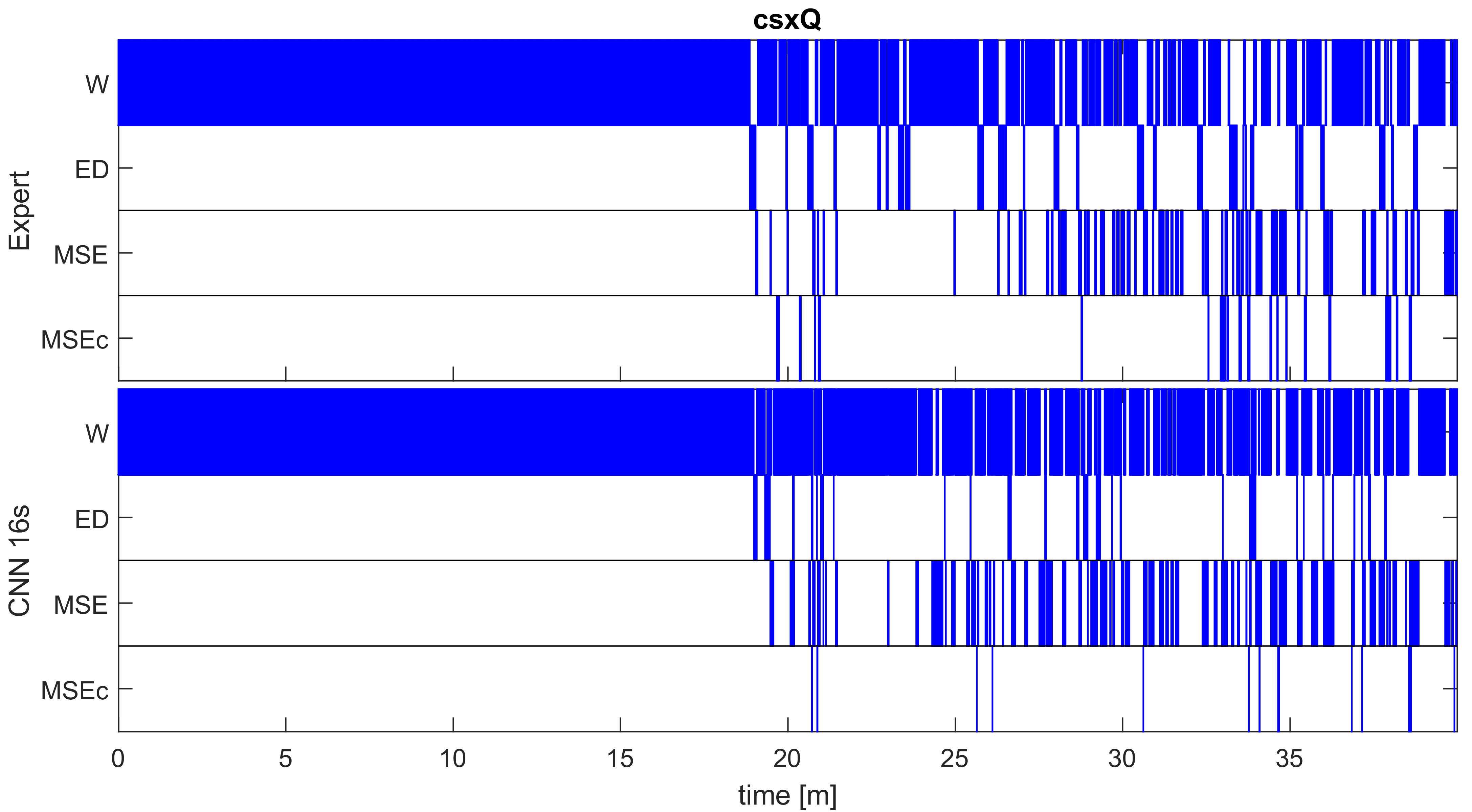 | 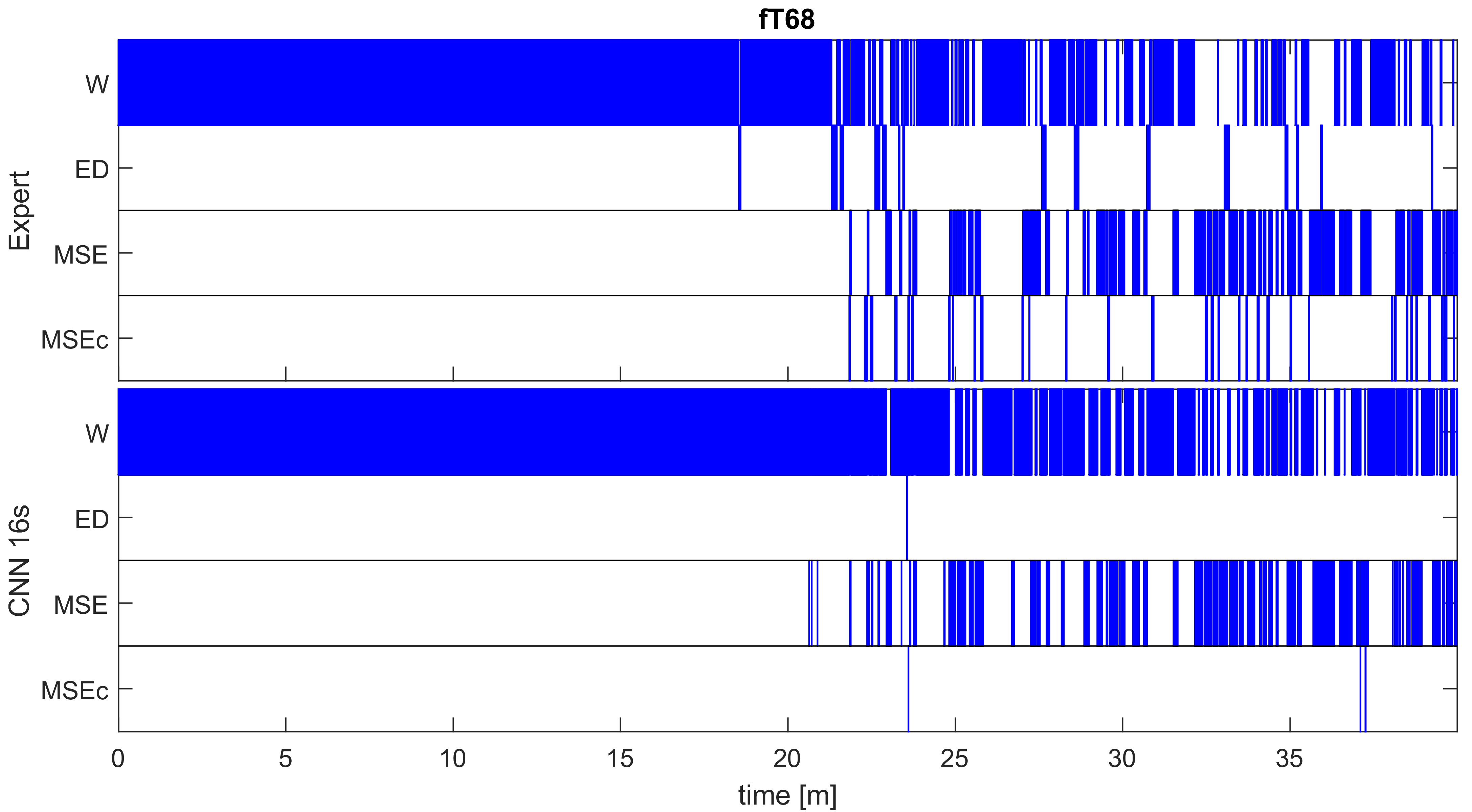 |
| 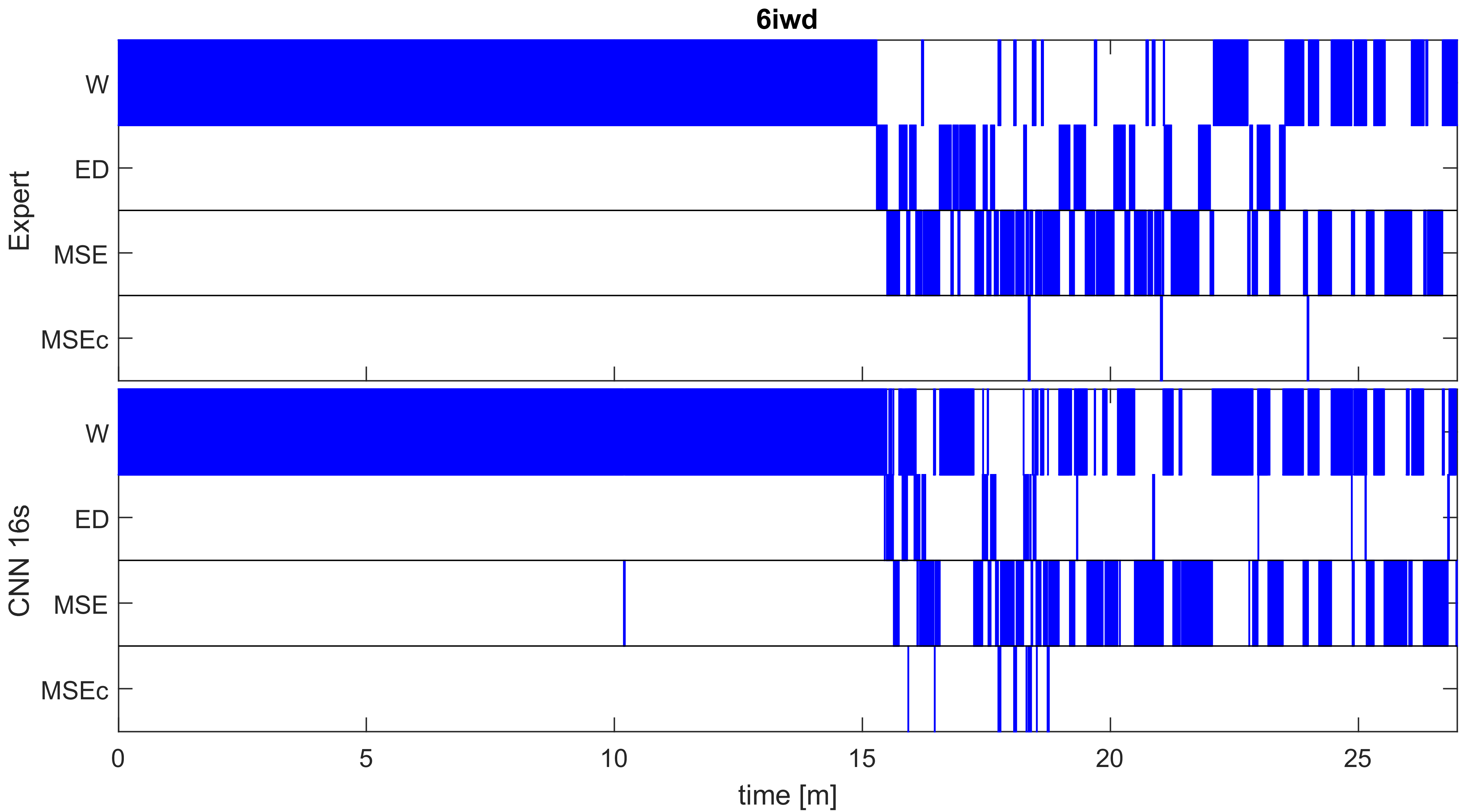 |  |

**Supplementary Figure S3.** T-distributed stochastic neighbor embedding (t-SNE) was used to illustrate the 53 patients of the training dataset and their classification mapped into a 2D space (last layer of the CNN 16s; arbitrary units). As to be expected for training data, all stages form clearly separated clusters except for very few data points. Wakefulness (W): blue; microsleep episodes (MSE): red; microsleep episode candidates (MSEc) green; episodes of drowsiness (ED): magenta. For the convenience we illustrated only every hundredth datapoint (sample). The patient ID is provided at the top of the plot. Please note that these figures only show the internal representation of the data in our specific network. Validation data are illustrated in Supplementary Figure S4.

| 6JVj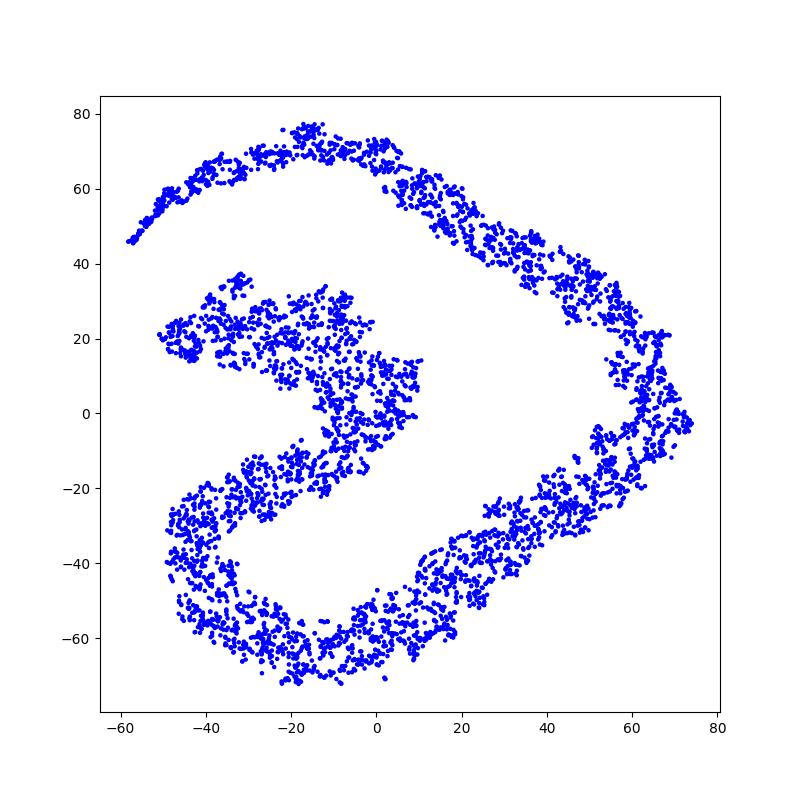 | 40kO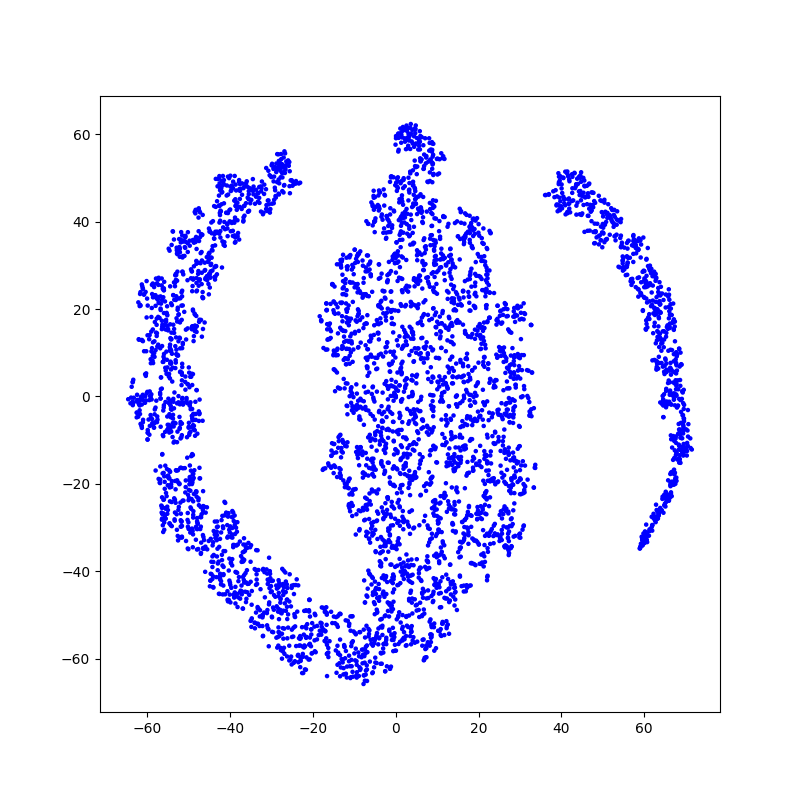 |
| --- | --- |
| 9098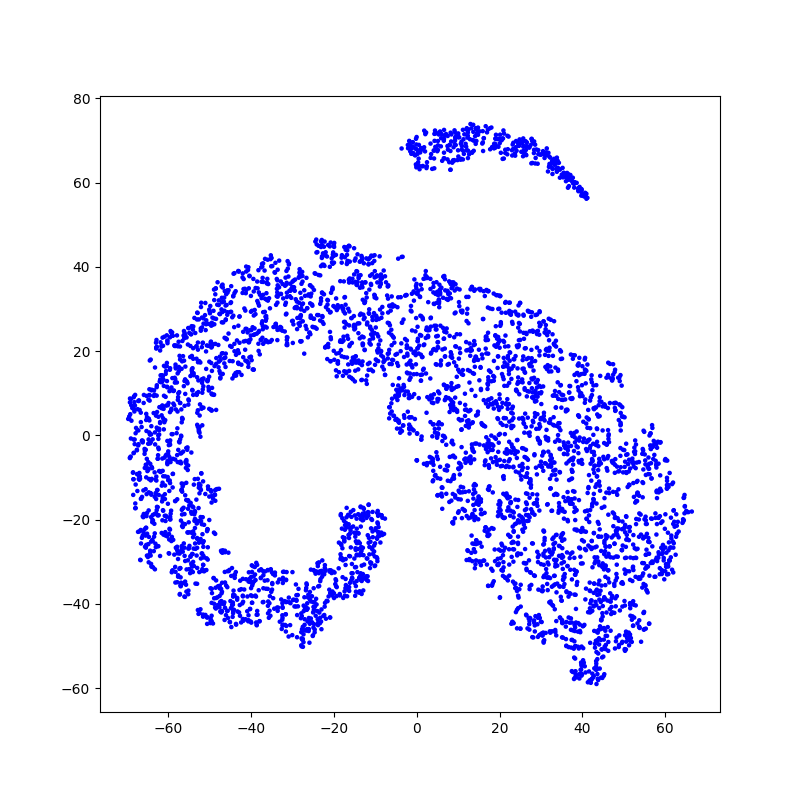 | AXbm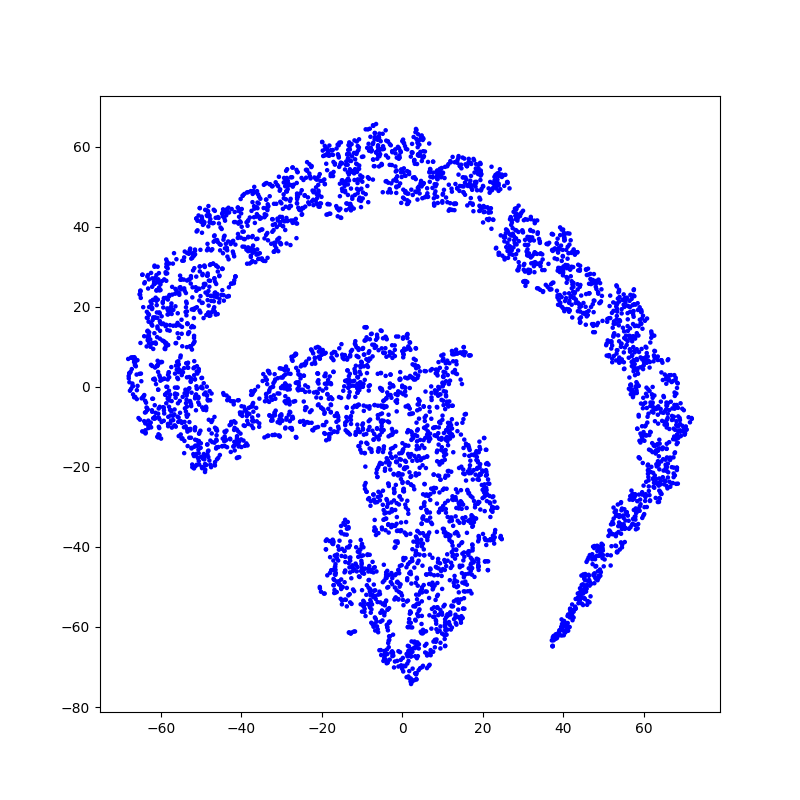 |

| cblr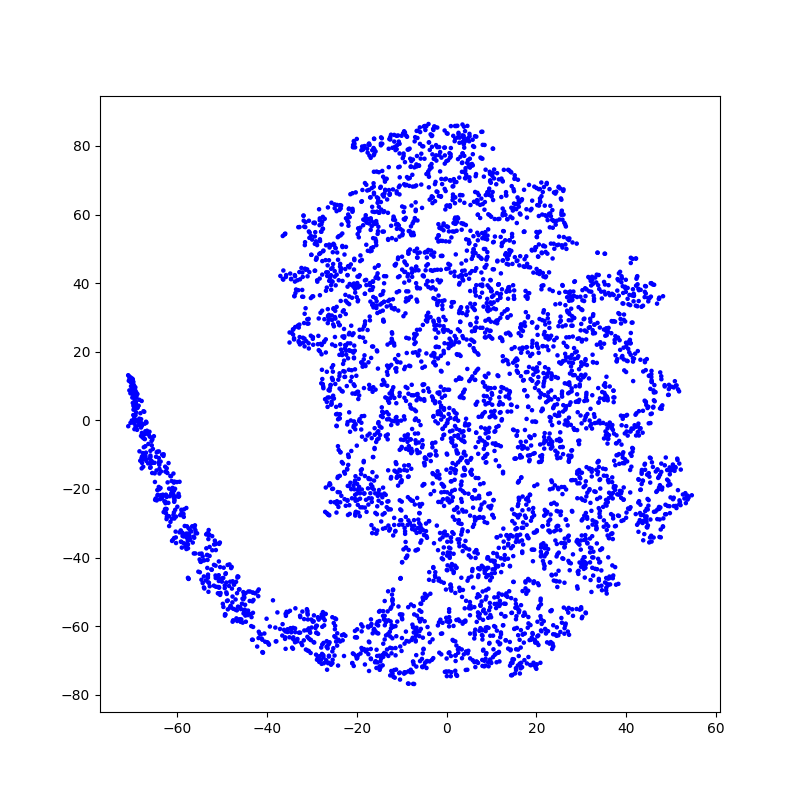 | DSfb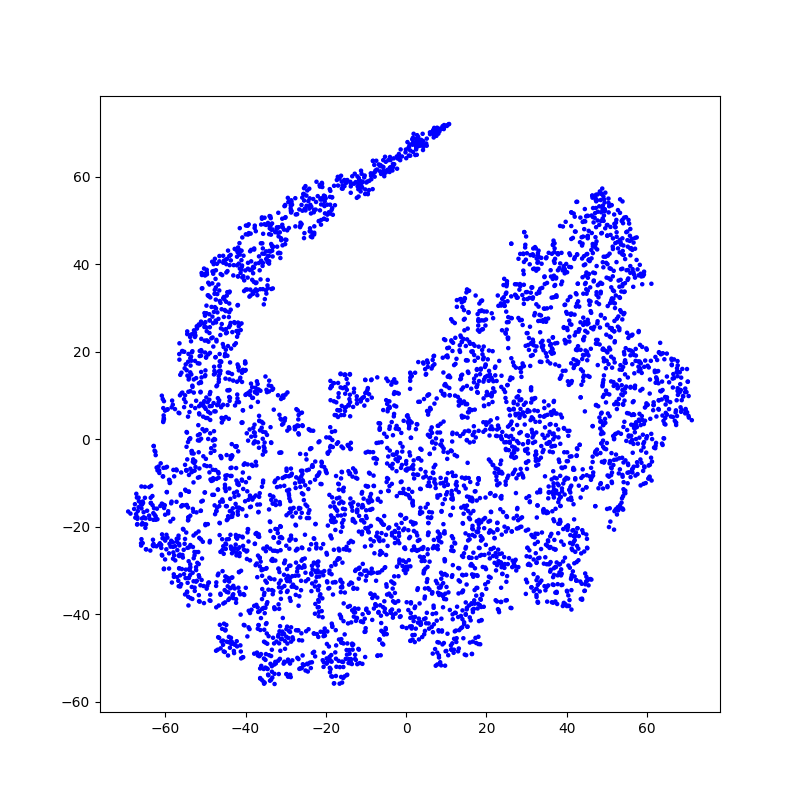 |
| --- | --- |
| EyTS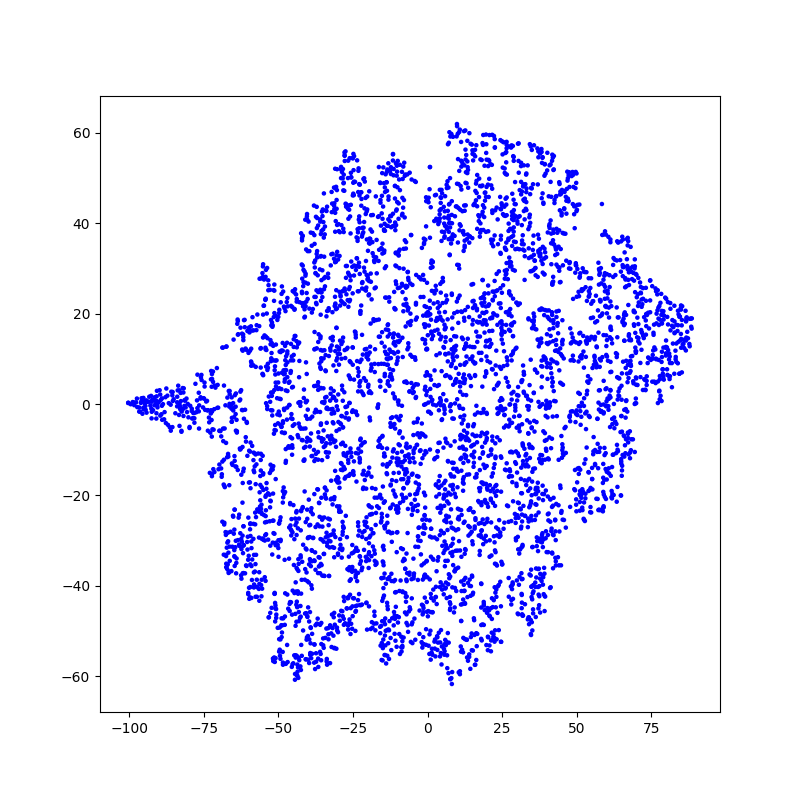 | mBks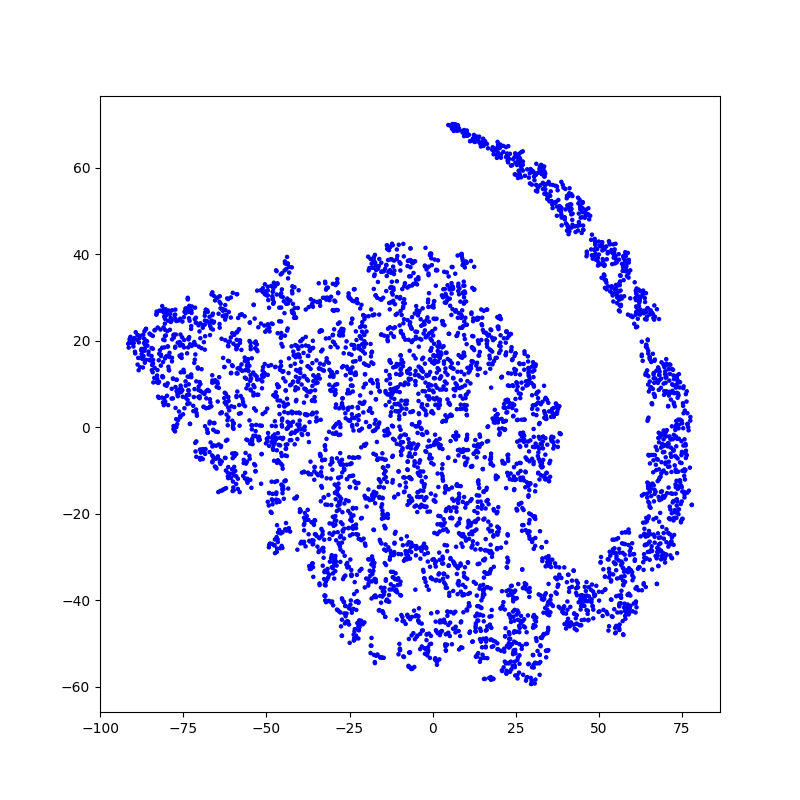 |
| fNe4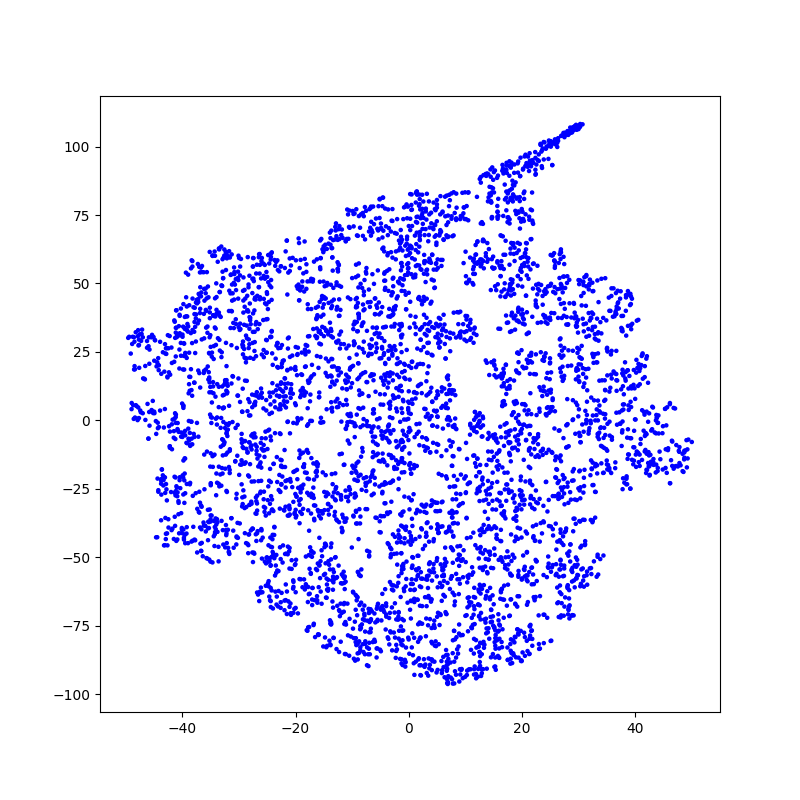 | go56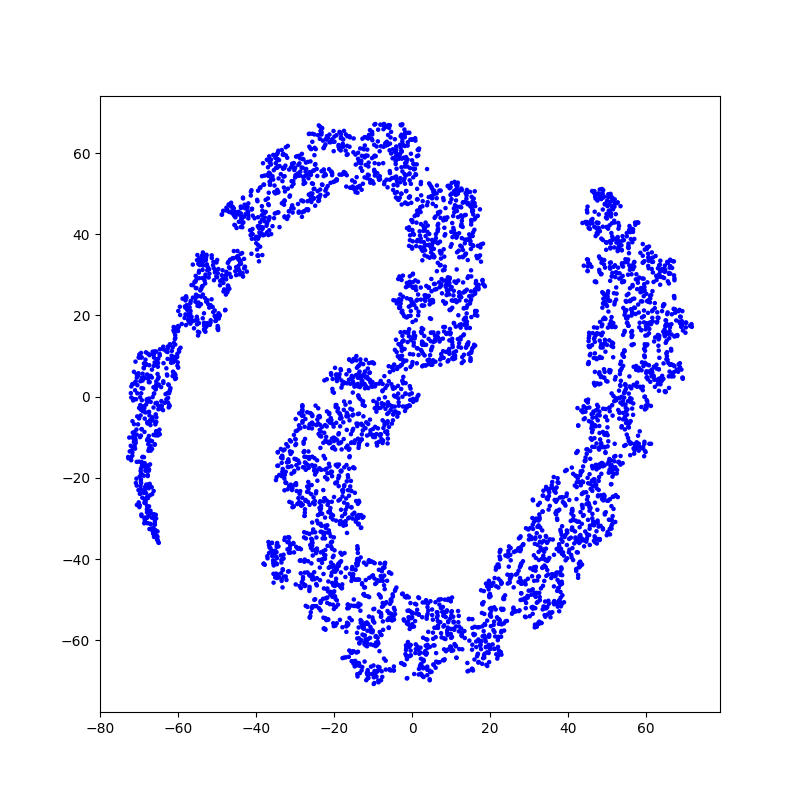 |
| hcml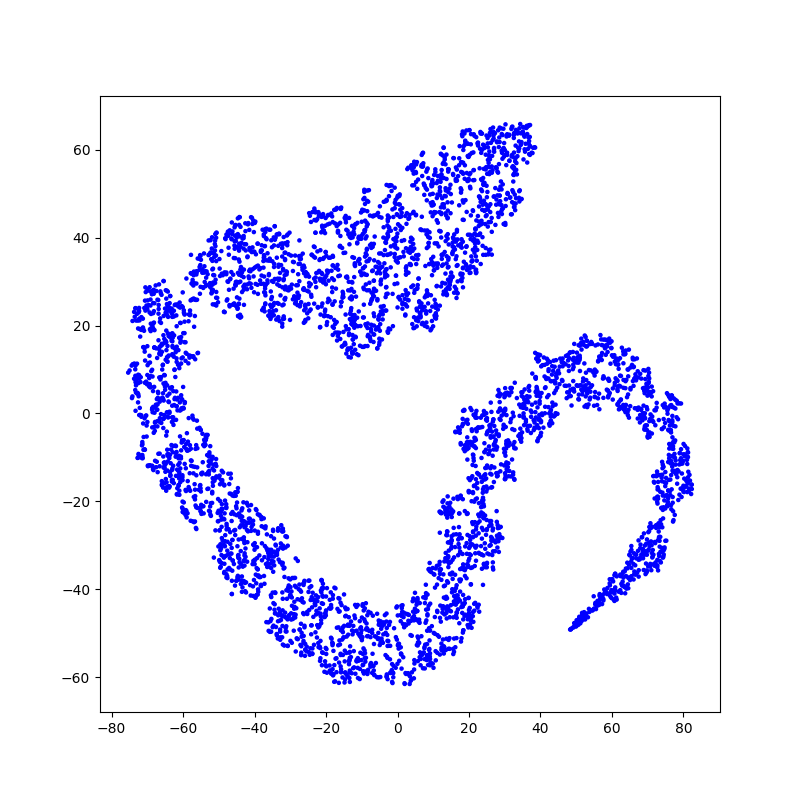 | hRMy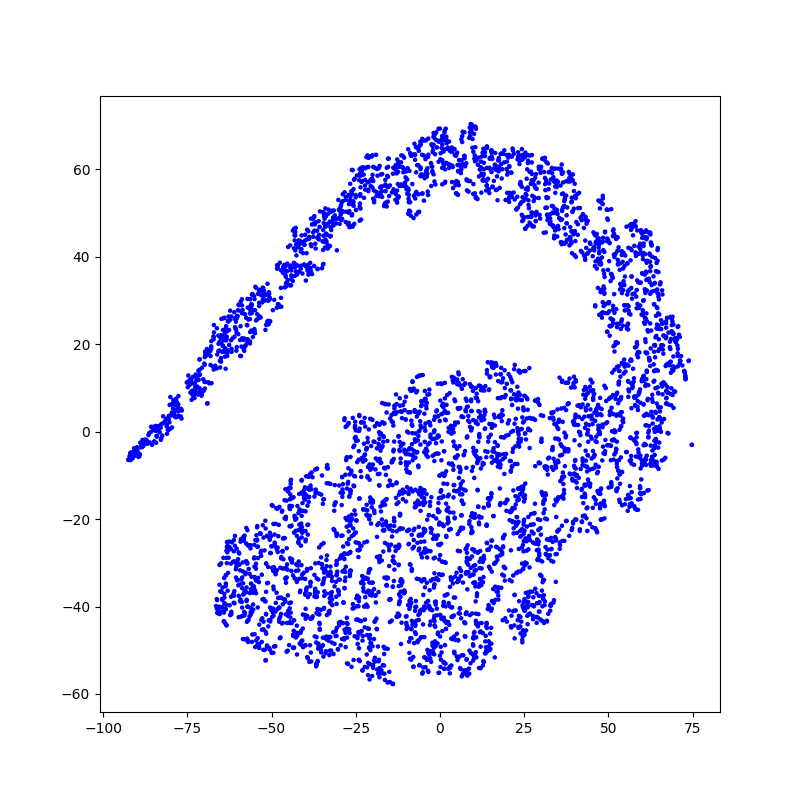 |
| muls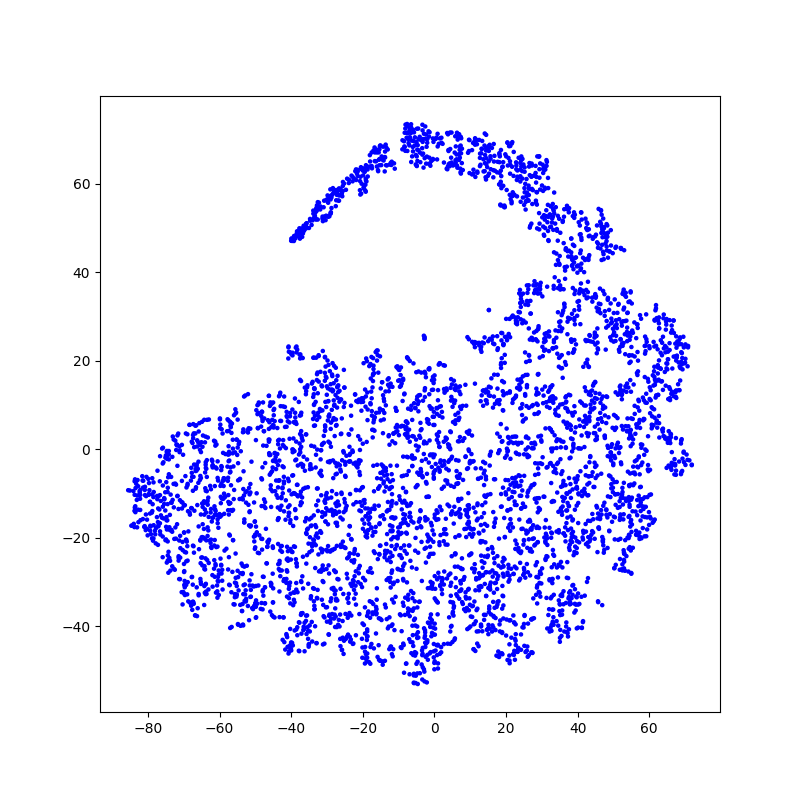 | mZje 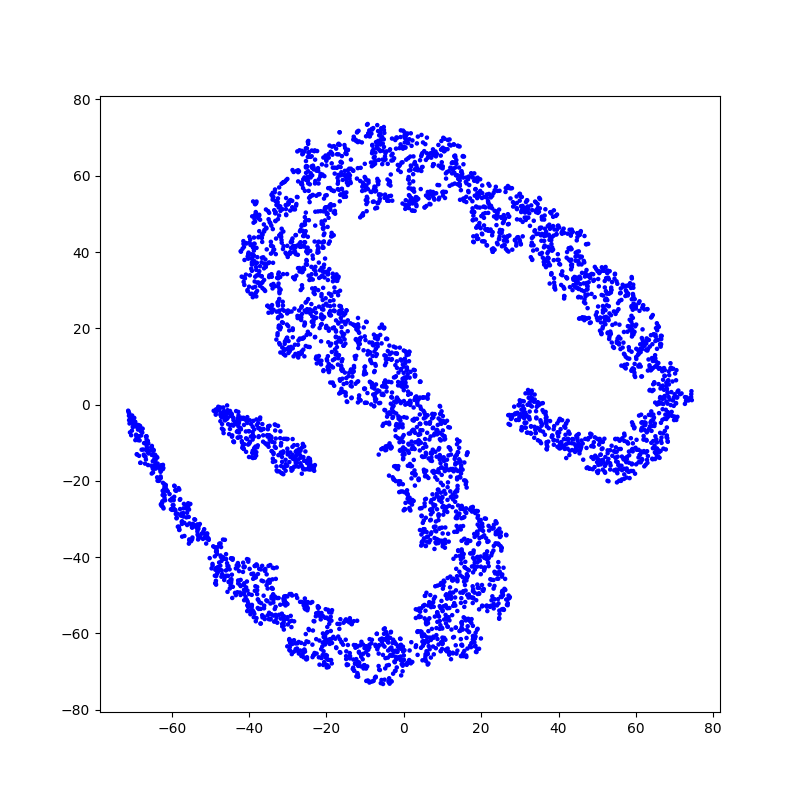 |
| UsSz 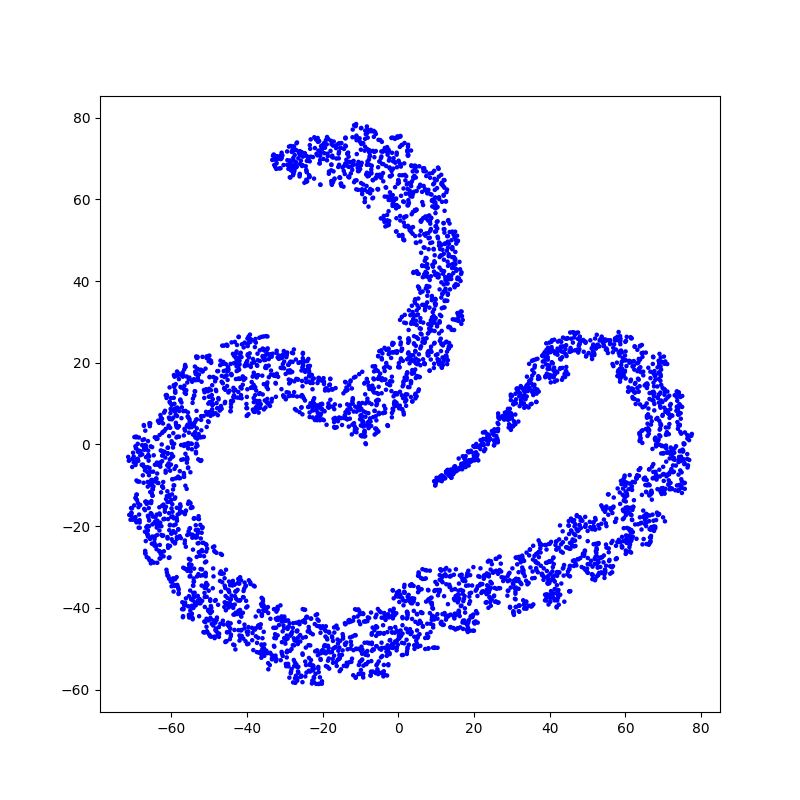 | YOh8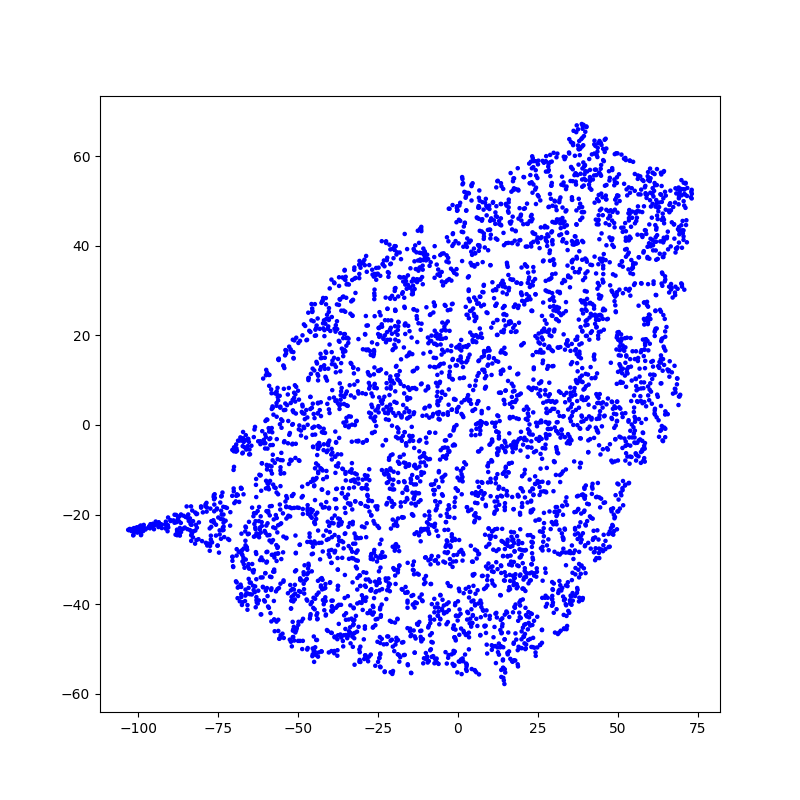 |
| 0ncr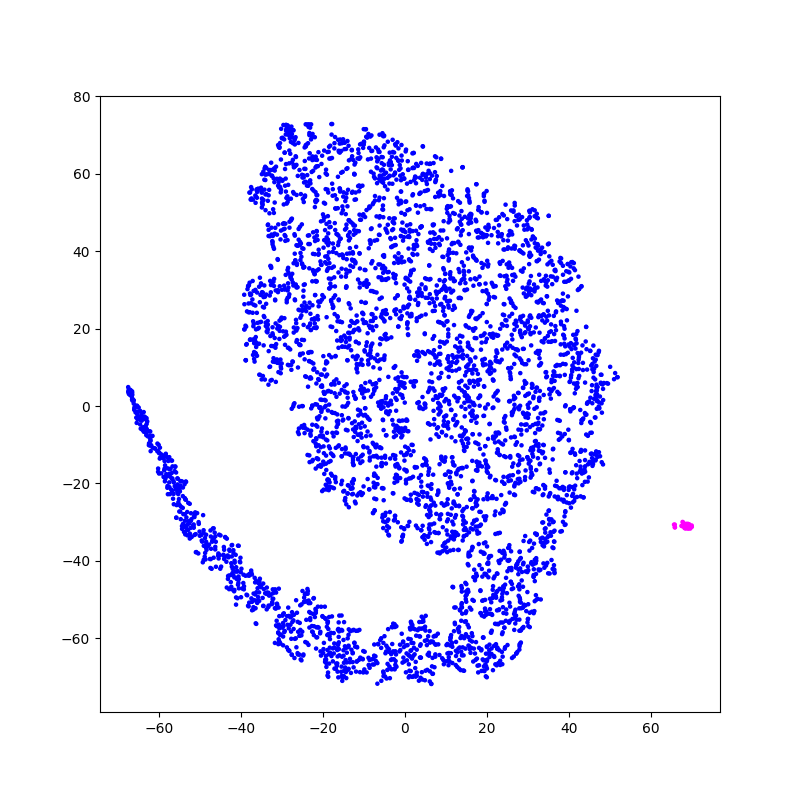 | MS6u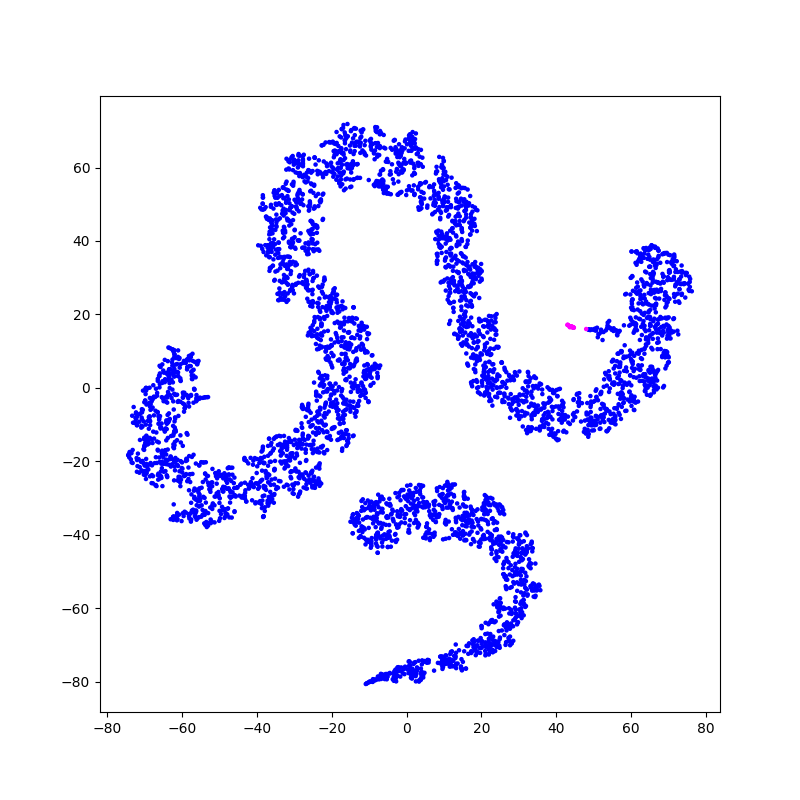 |
| pPpj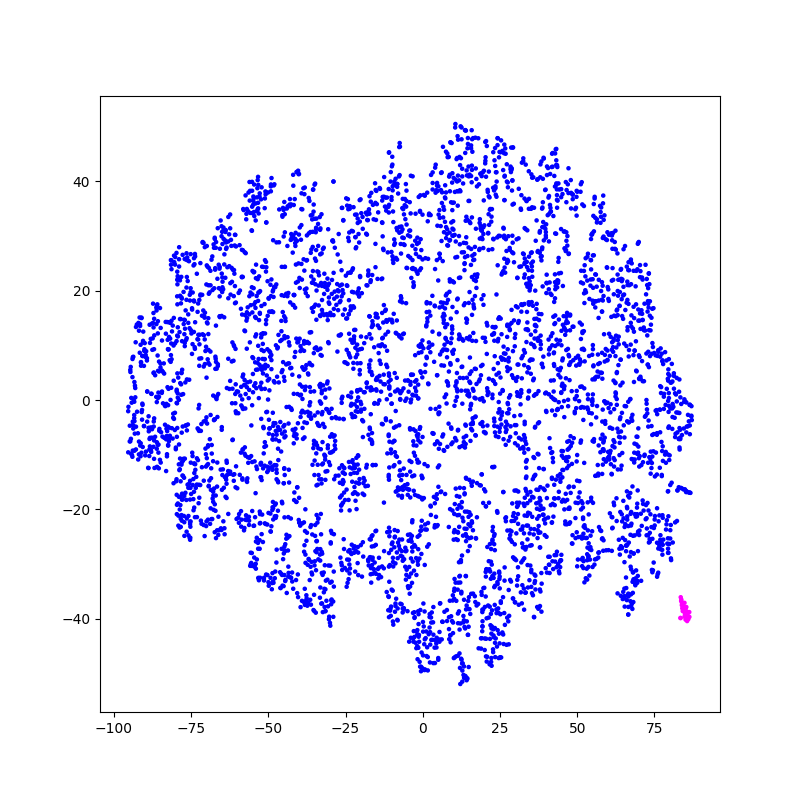 | UwK6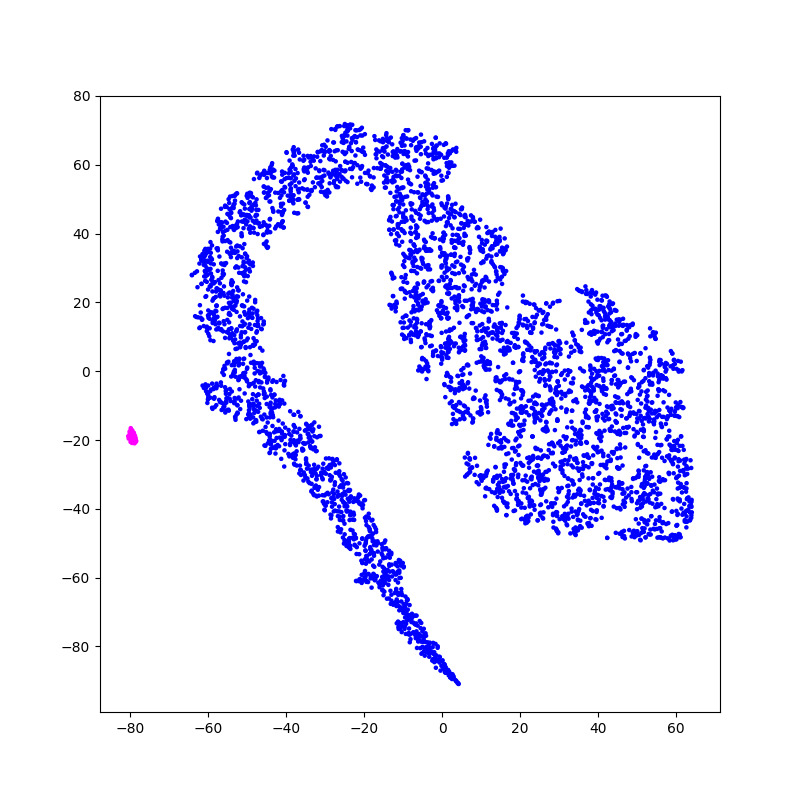 |
| Xii6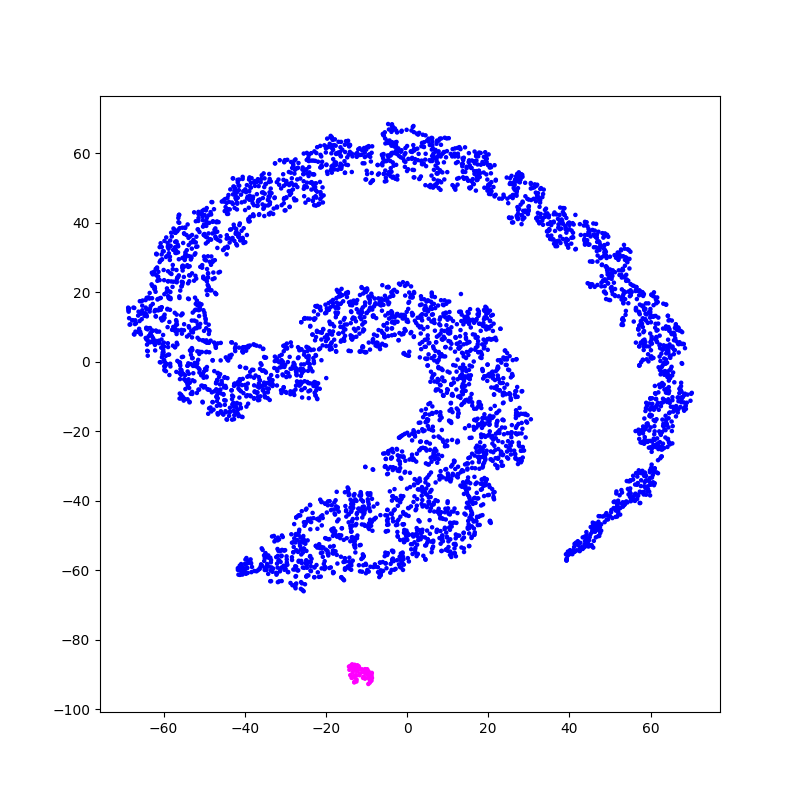 | YHLr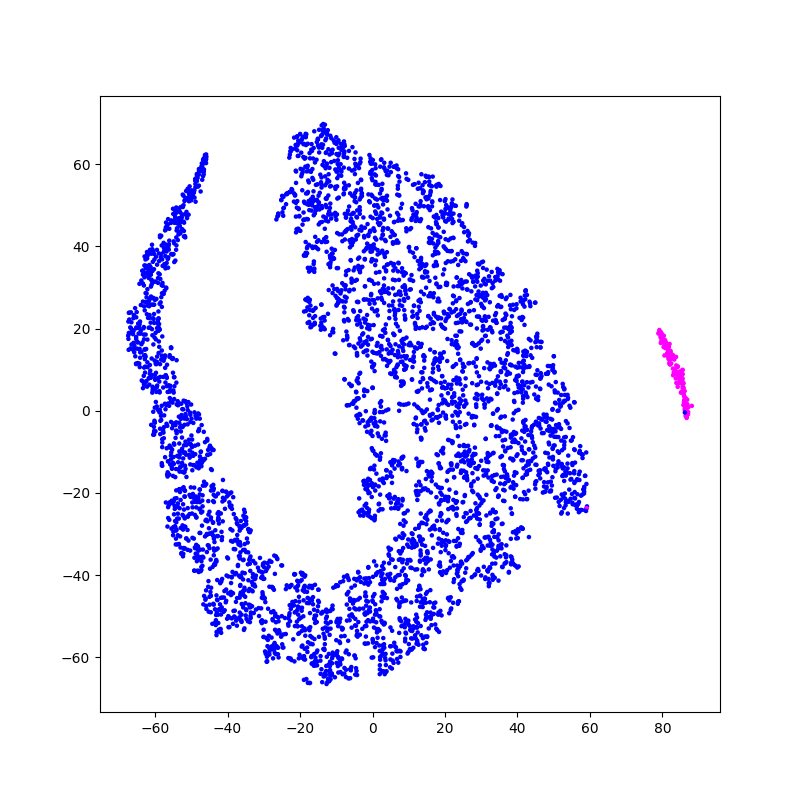 |
| EHED 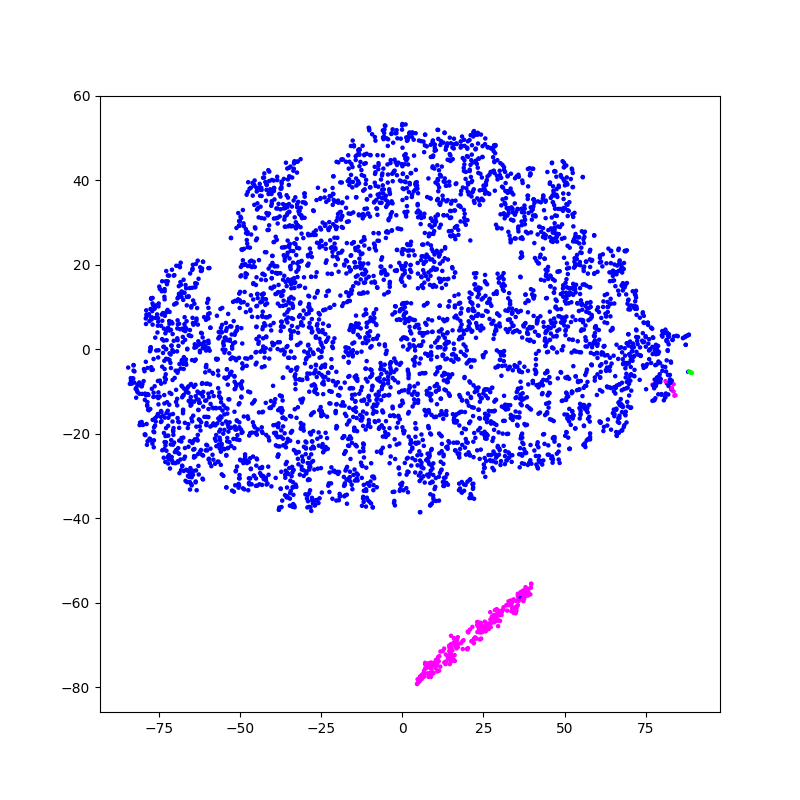 | Kn9O 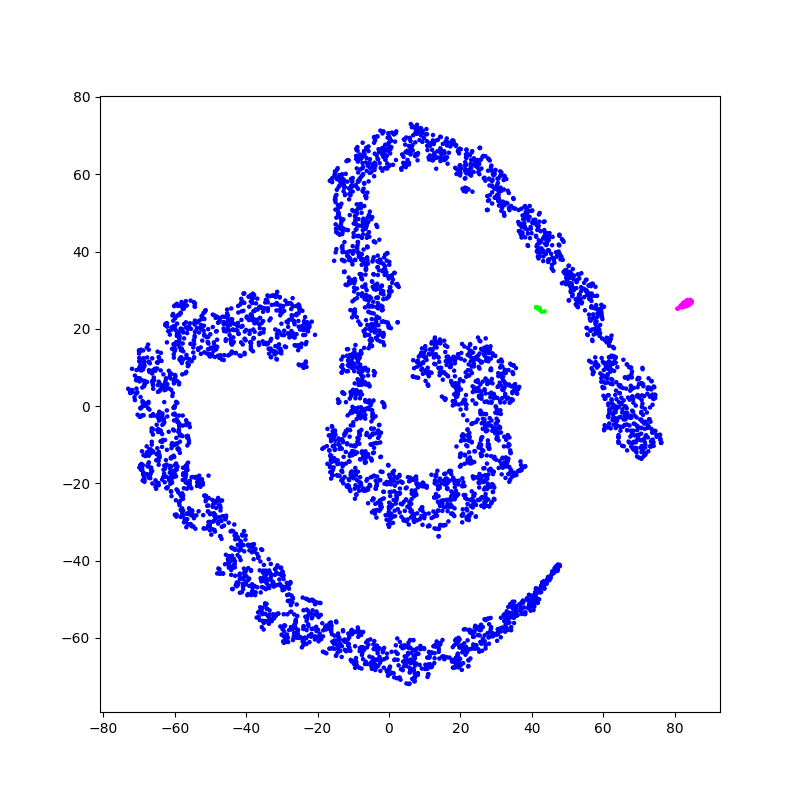 |
| Dr51 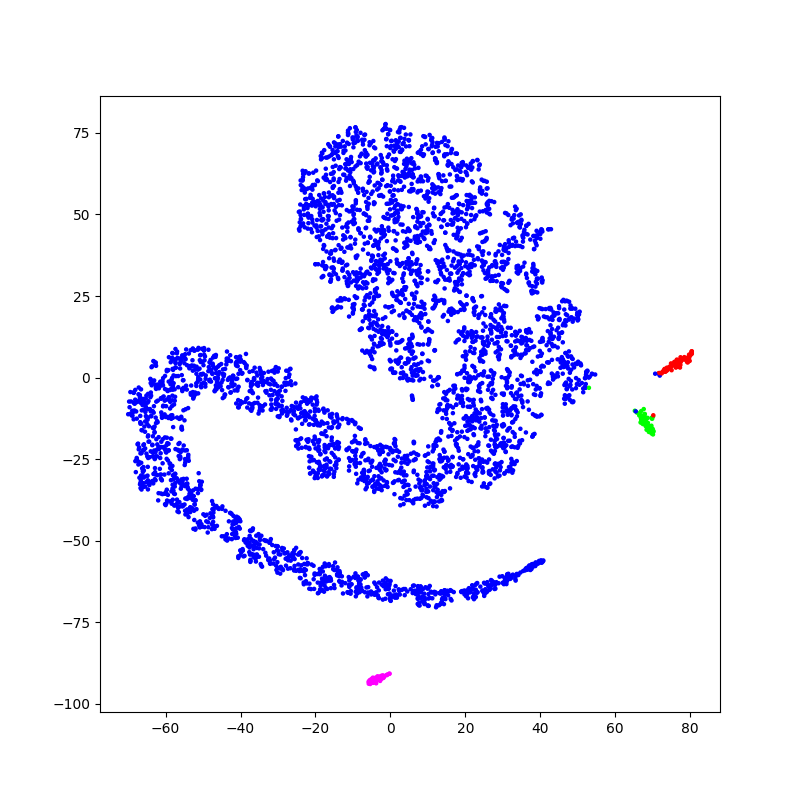 | DYYl 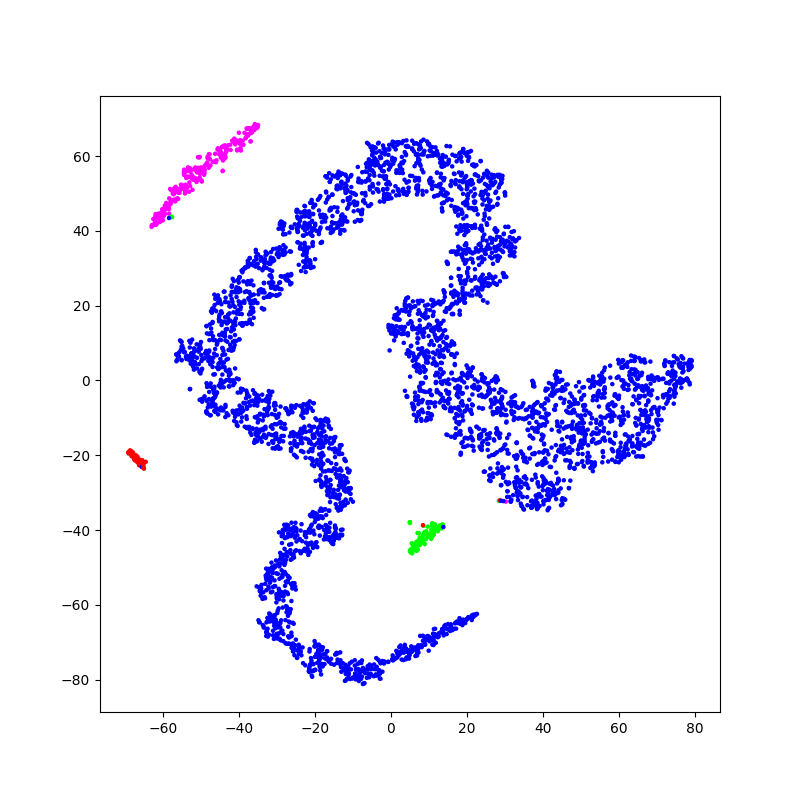 |
| kj2l 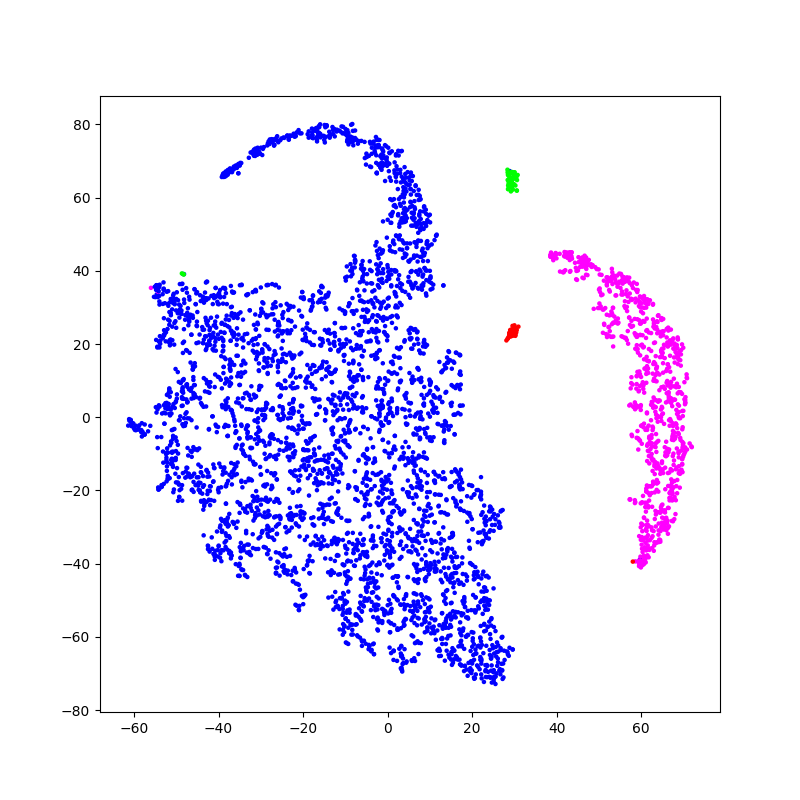 | LR2s 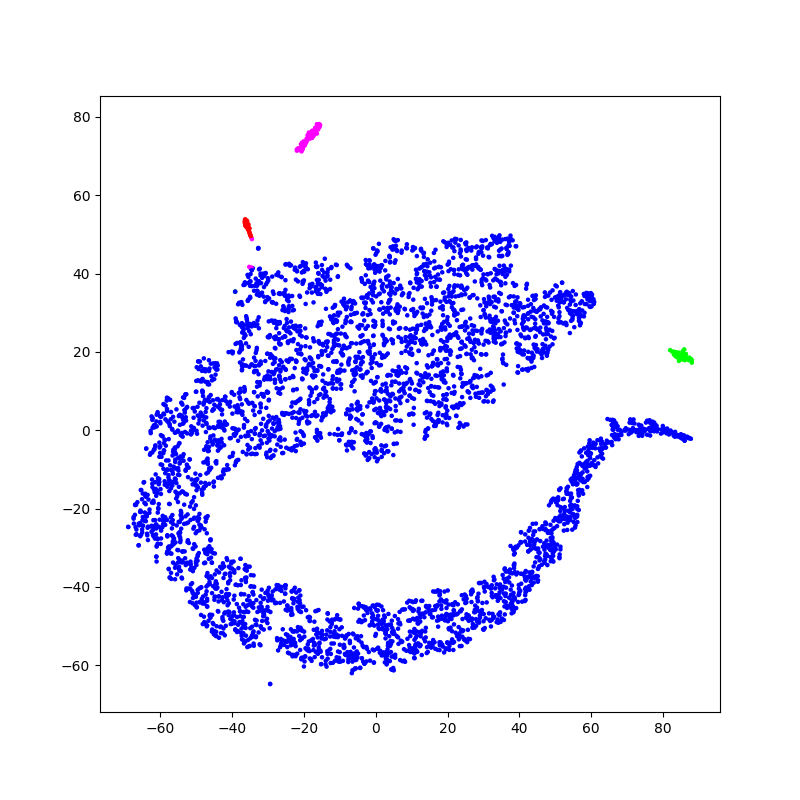 |
| SOZ3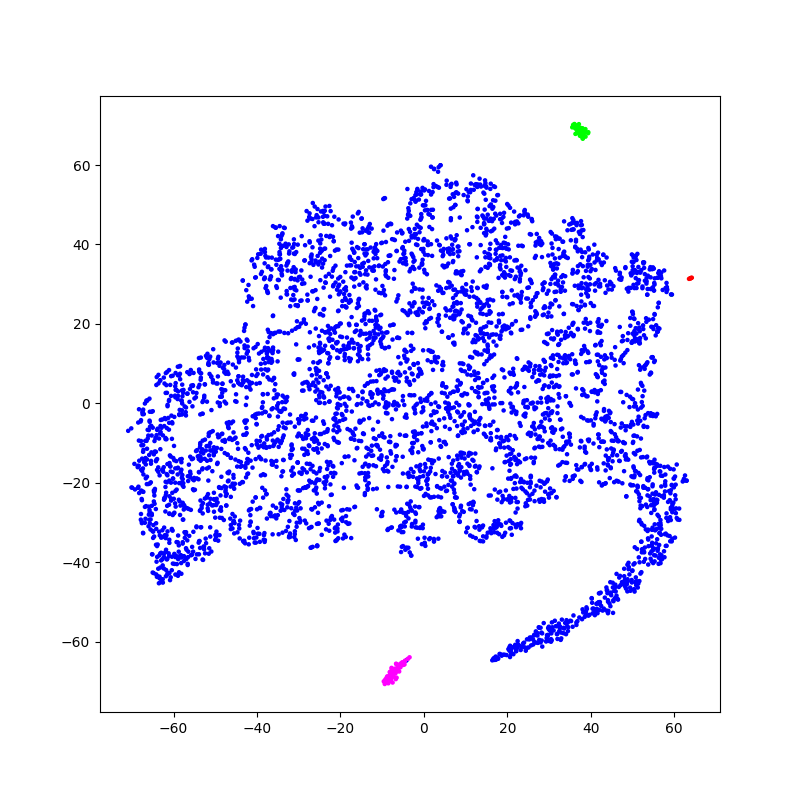 | Zpwh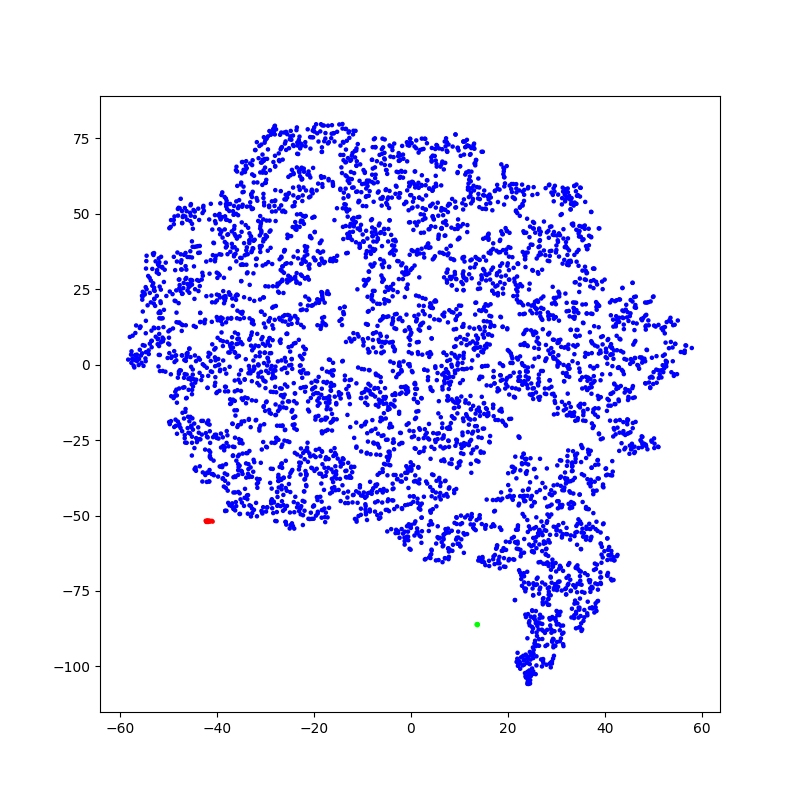 |
| 3P0D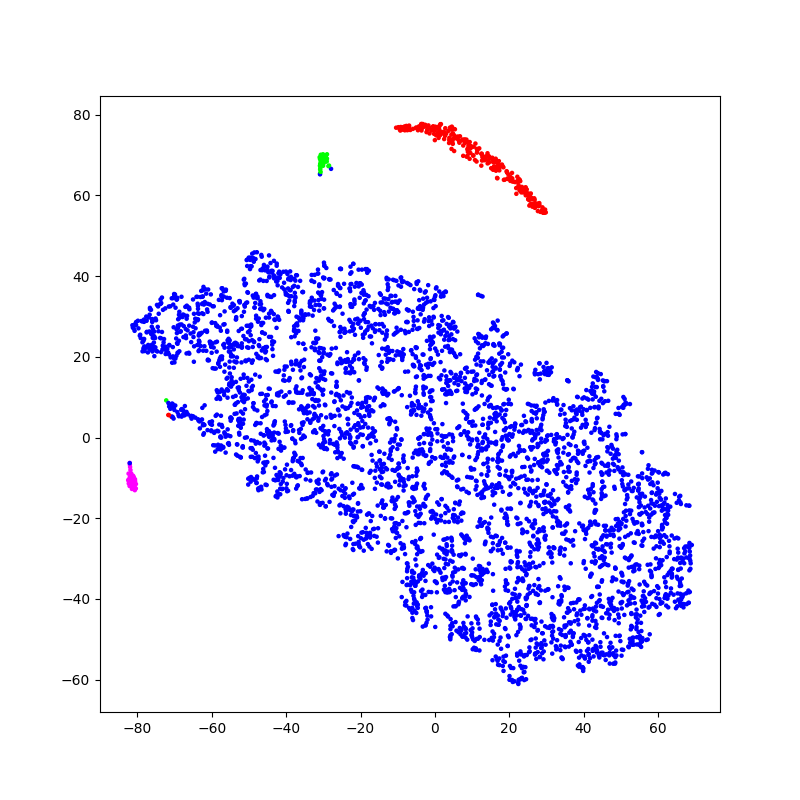 | bkx9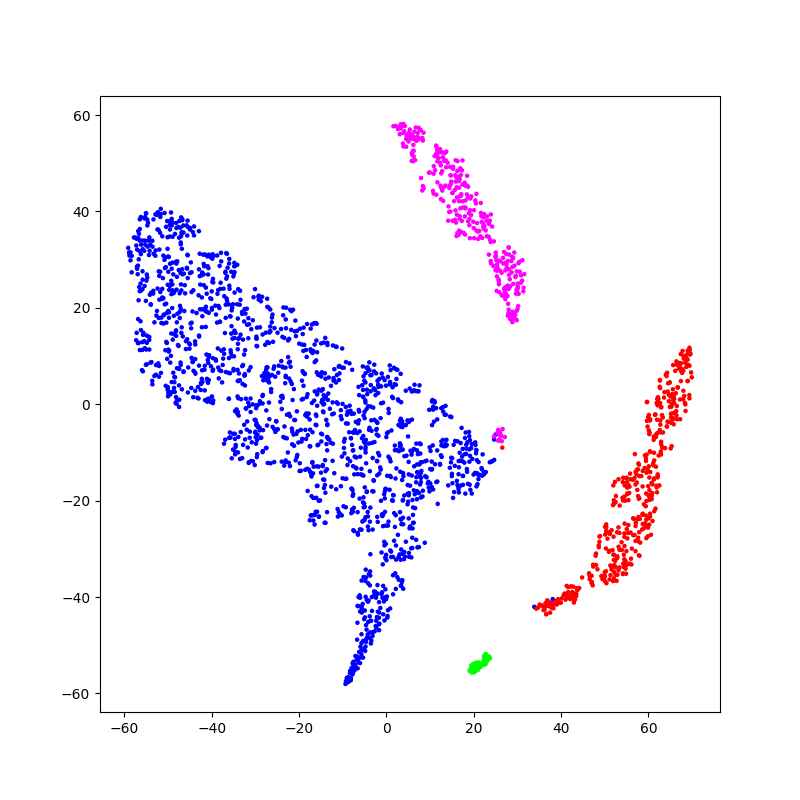 |
| C1Wu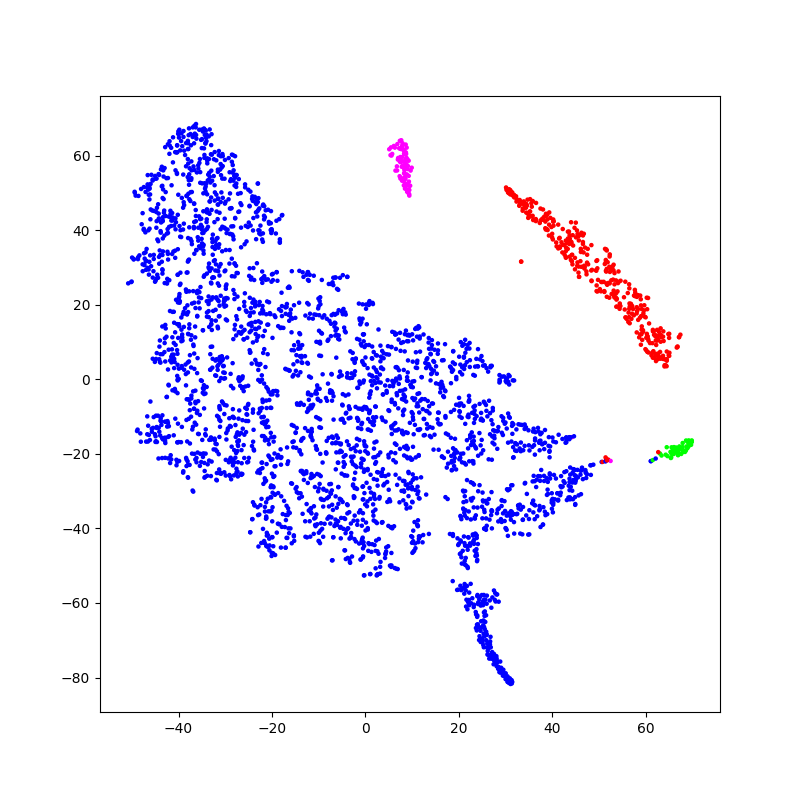 | DjrT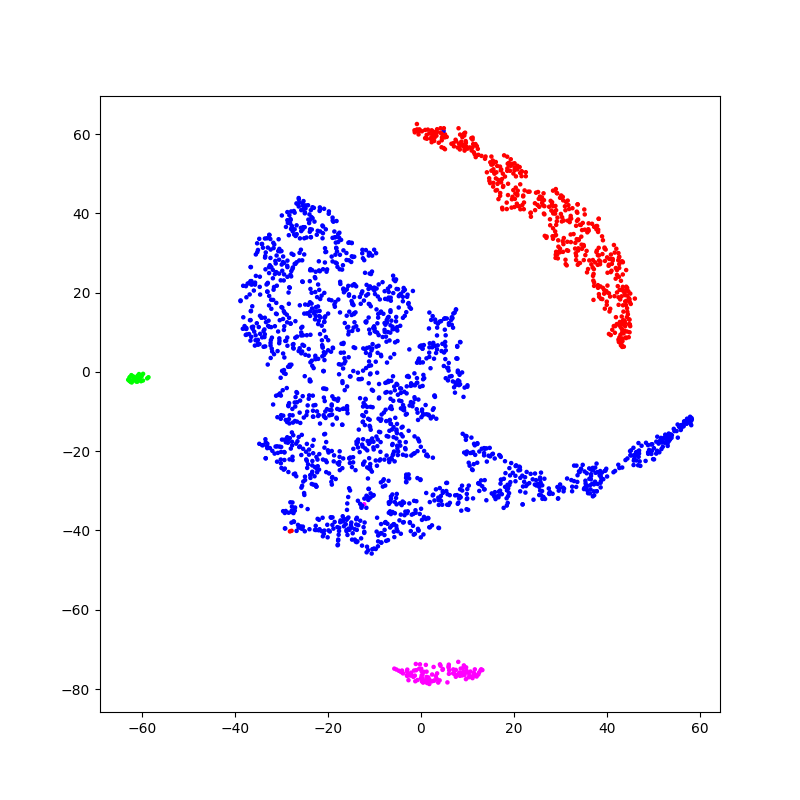 |
| EMcQ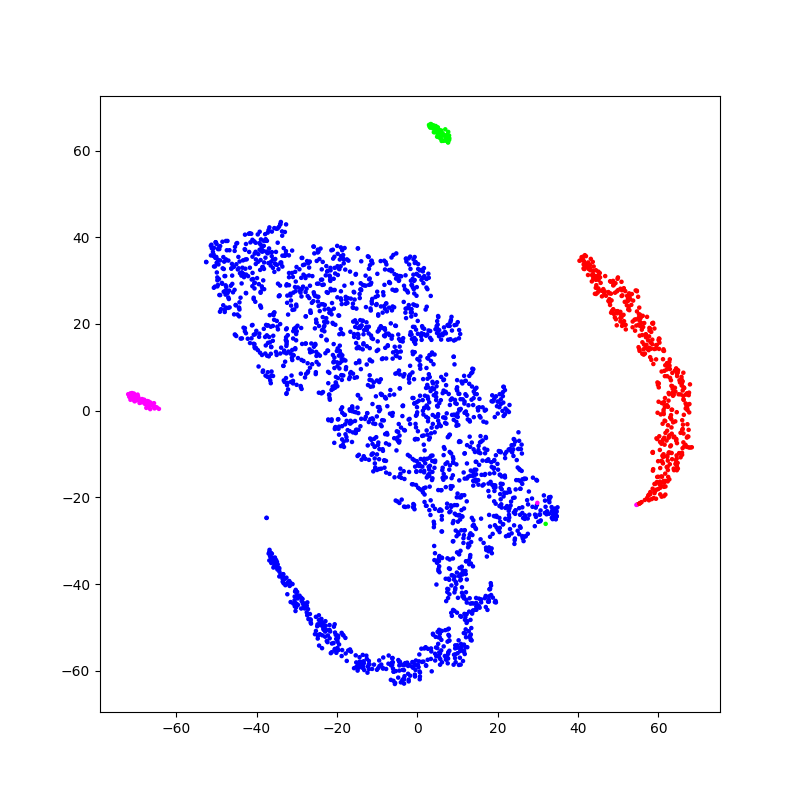 | hT38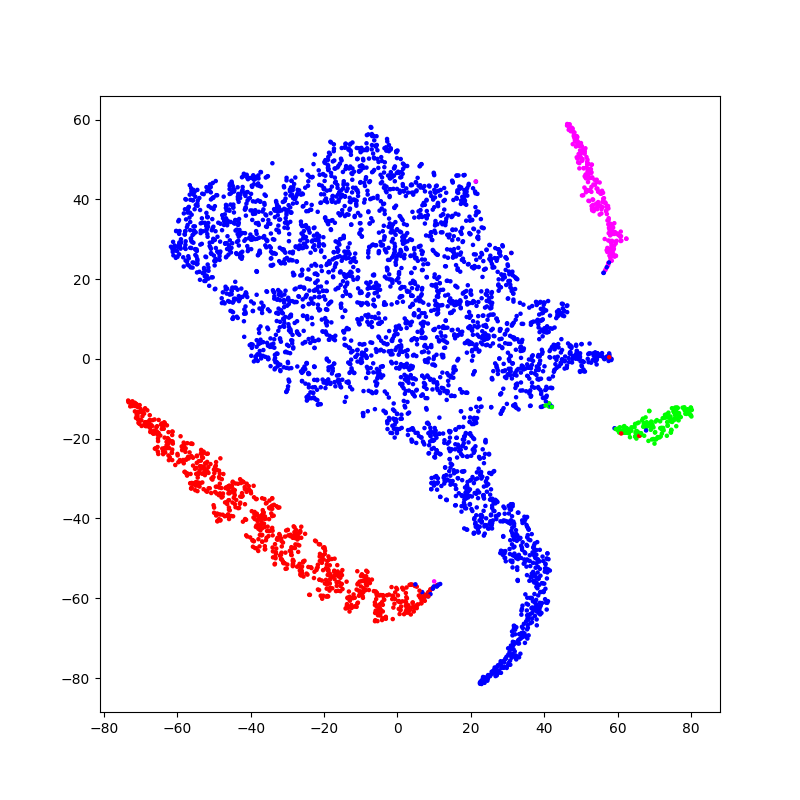 |
| ibbz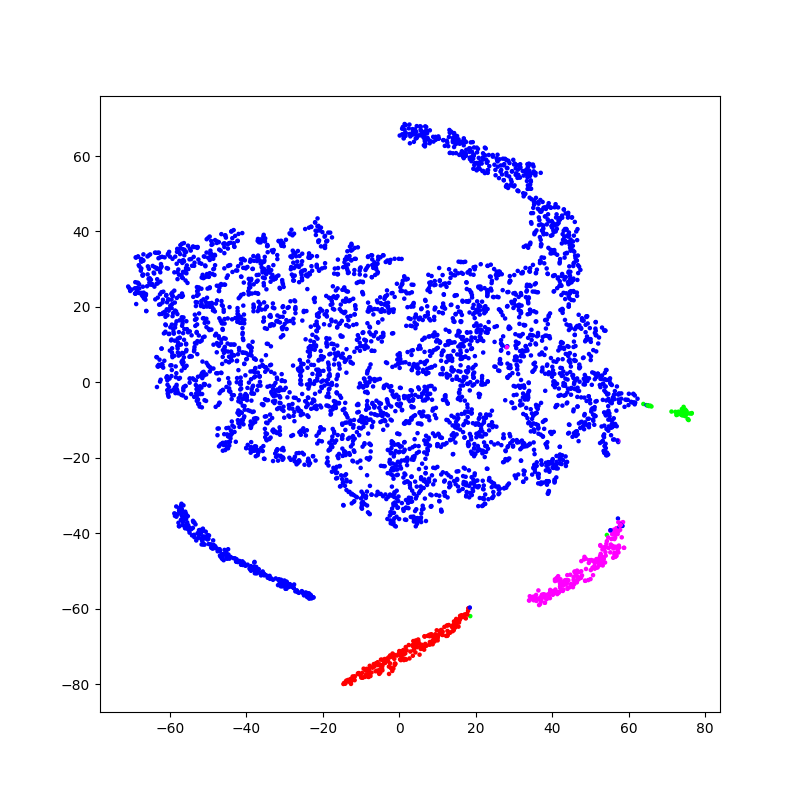 | iSqw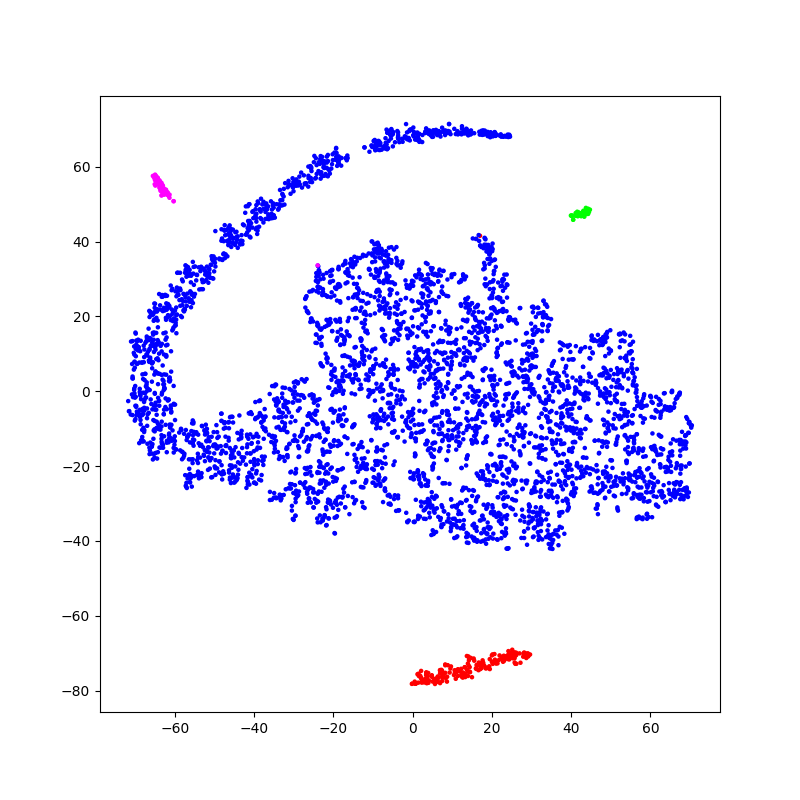 |
| Ivfn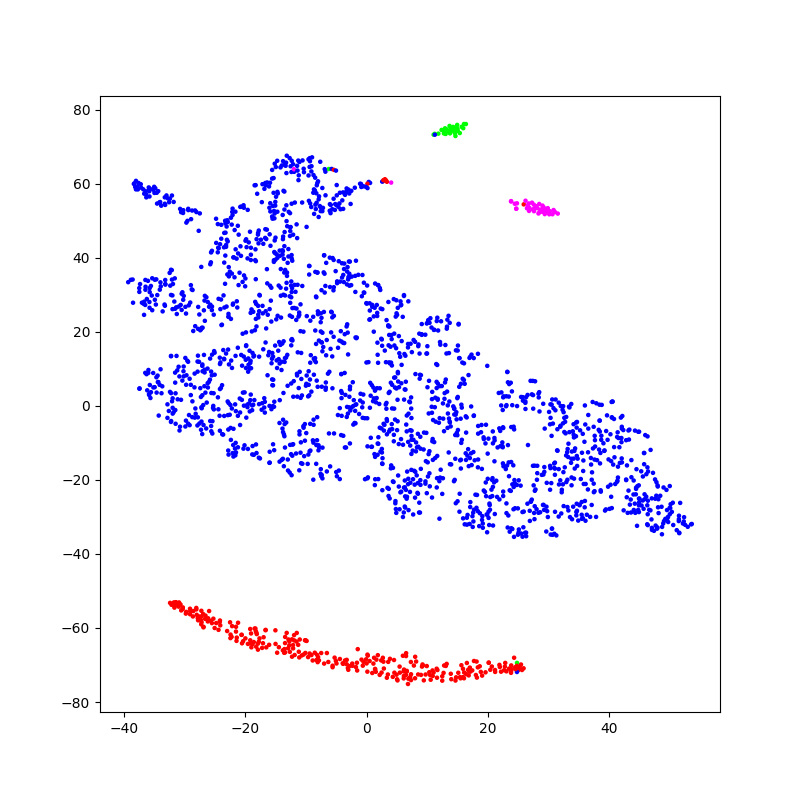 | Otq3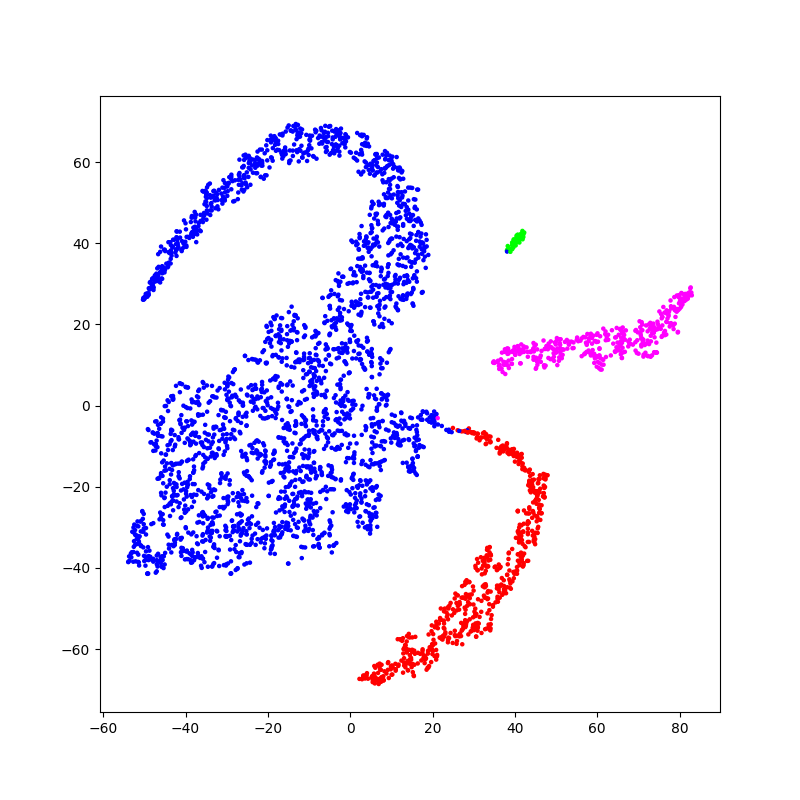 |
| tG6i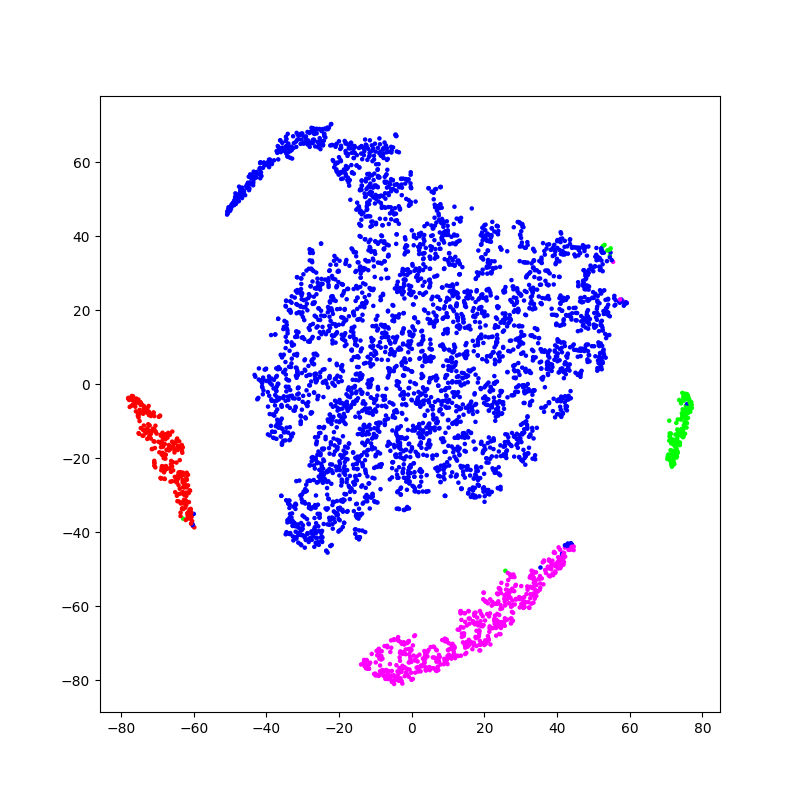 | X7s0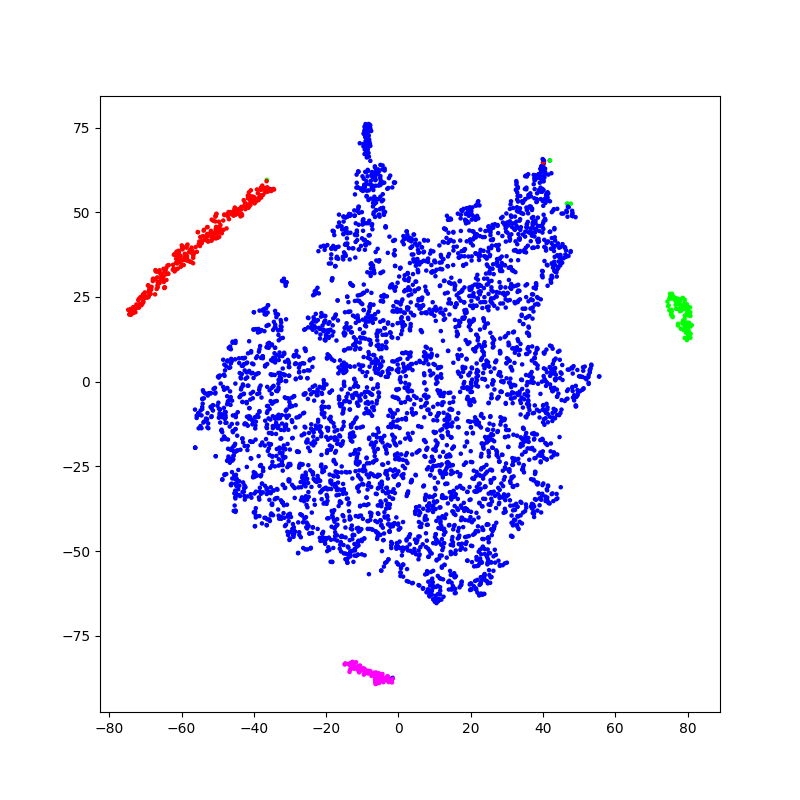 |
| ZYFG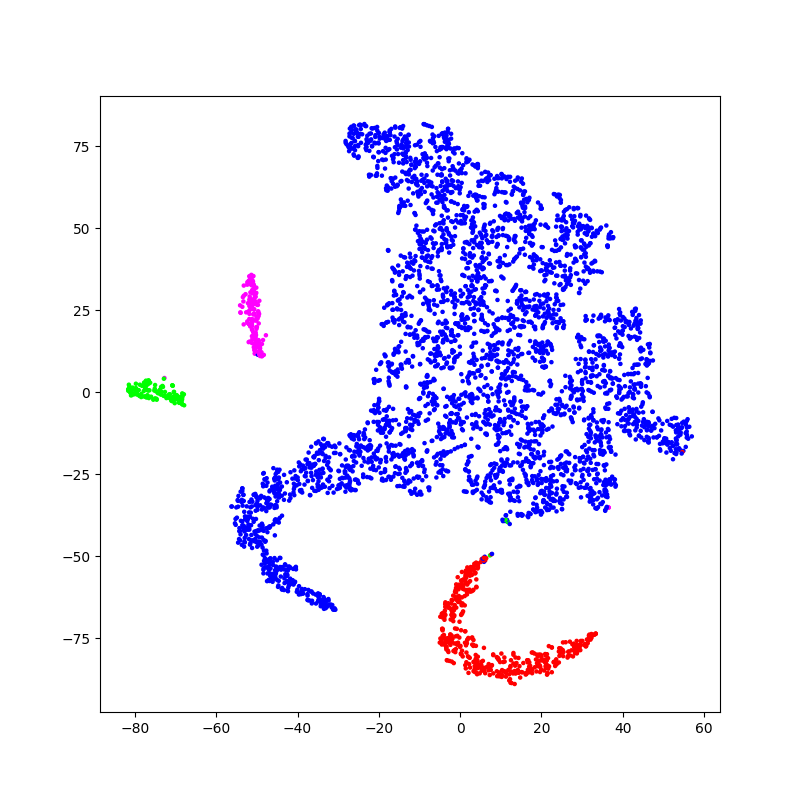 | 5bSg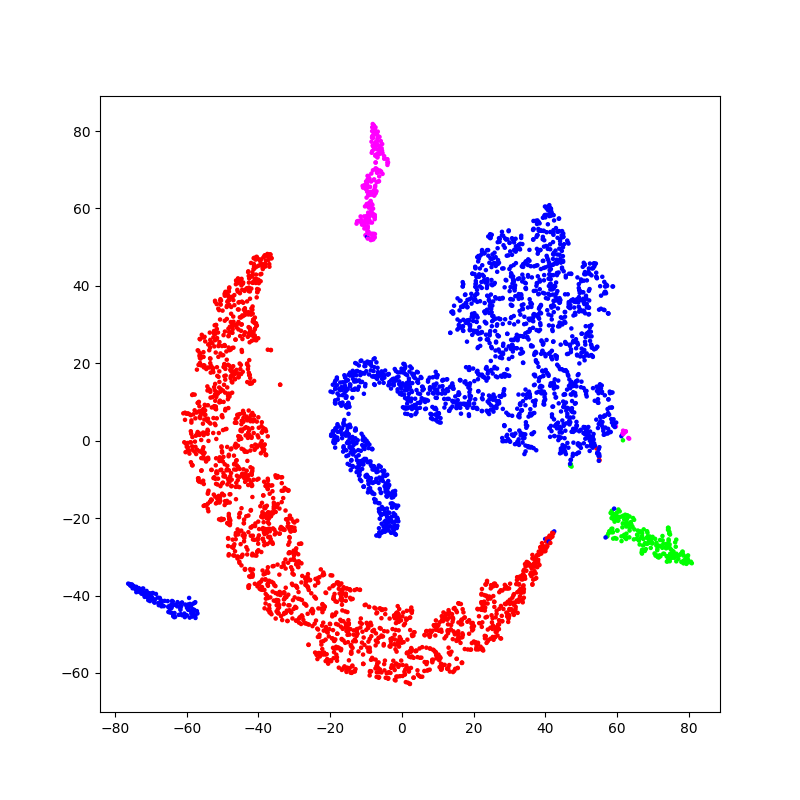 |
| BSvO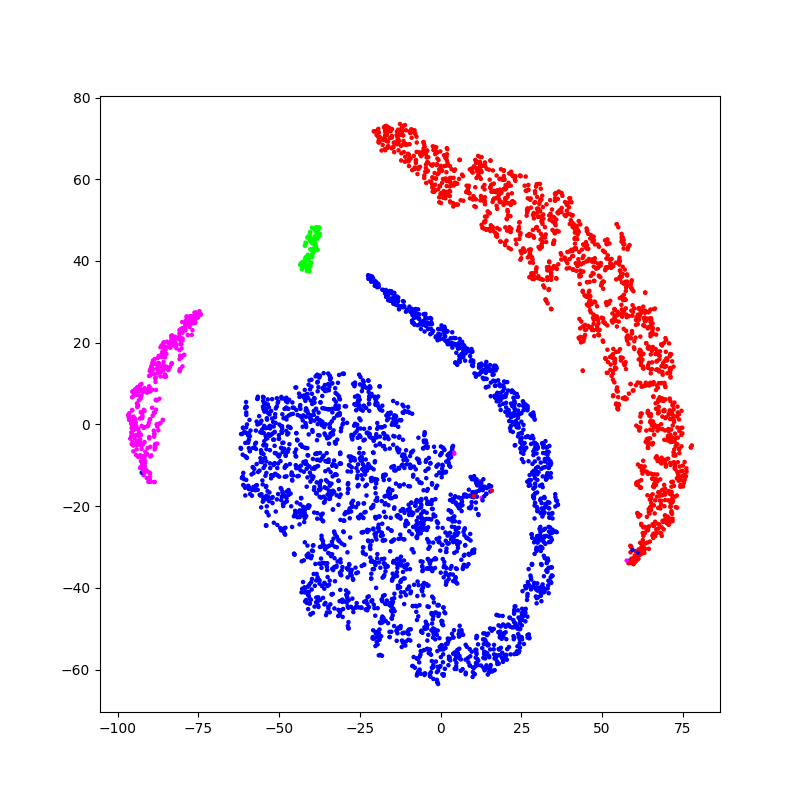 | G7PJ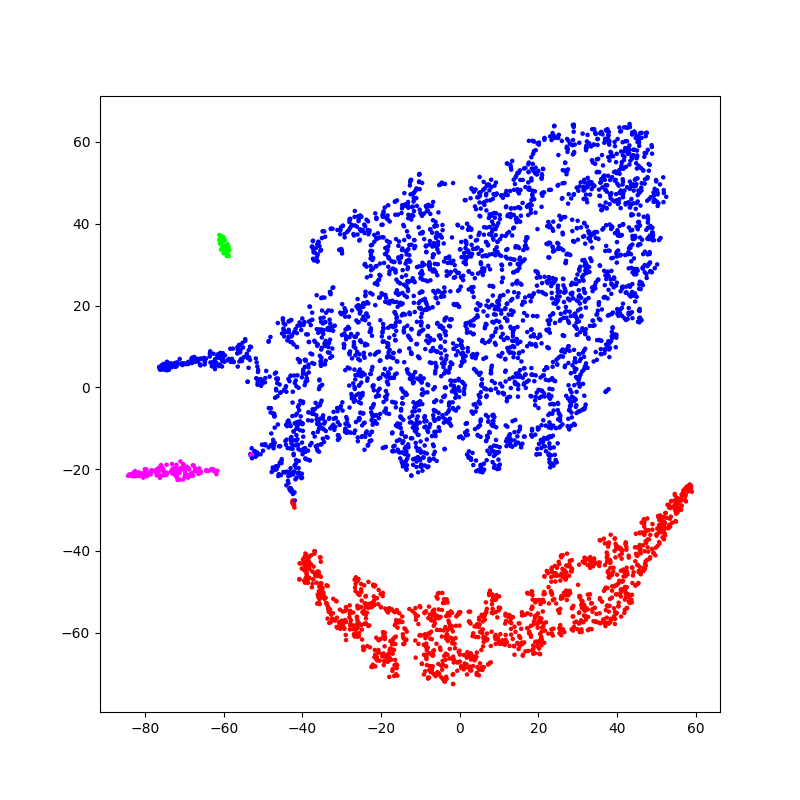 |
| lhpU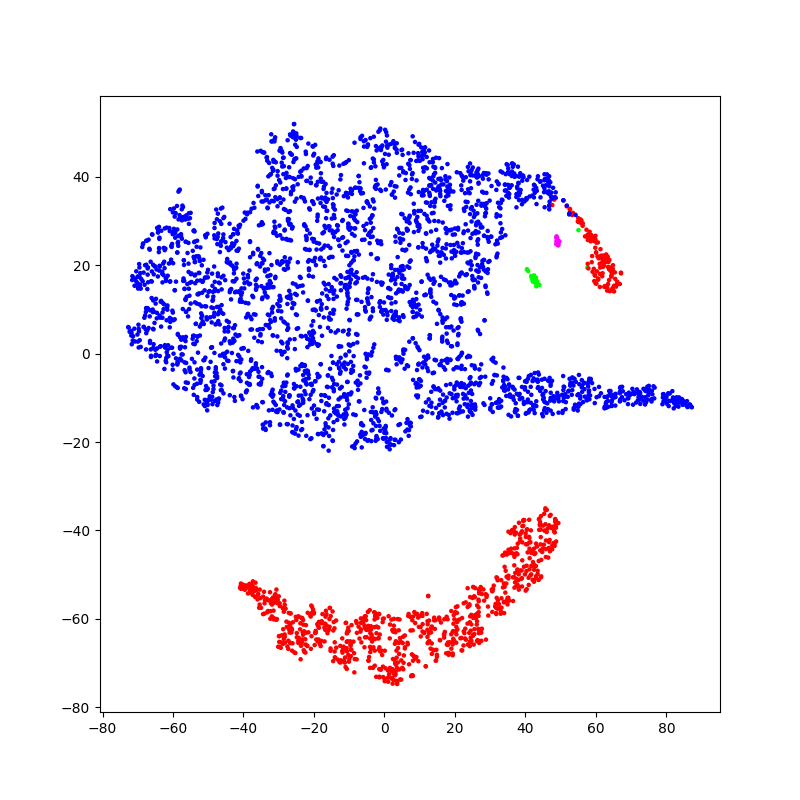 | Msy4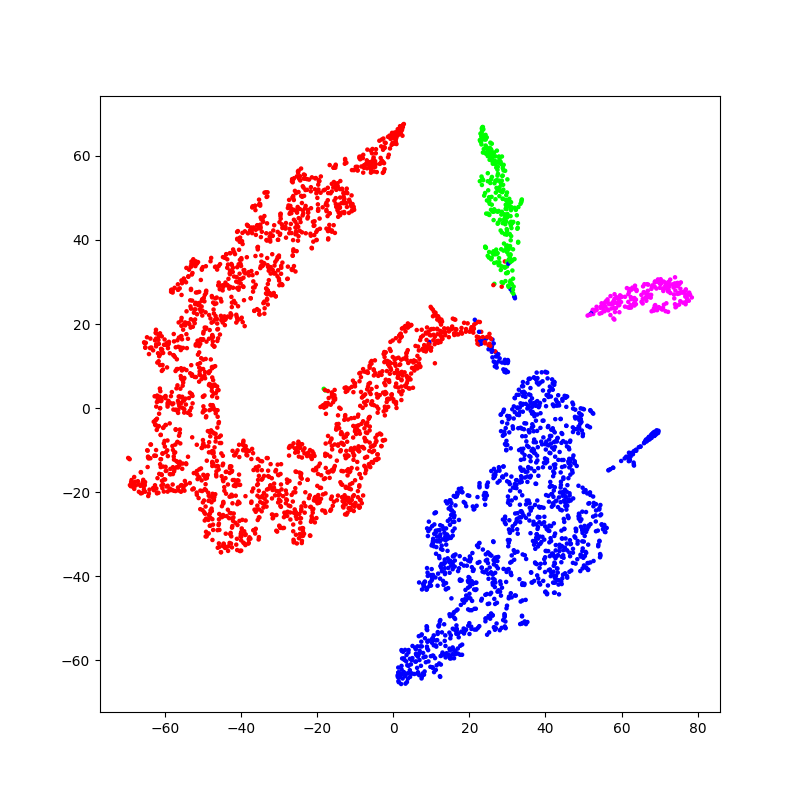 |
| N1nM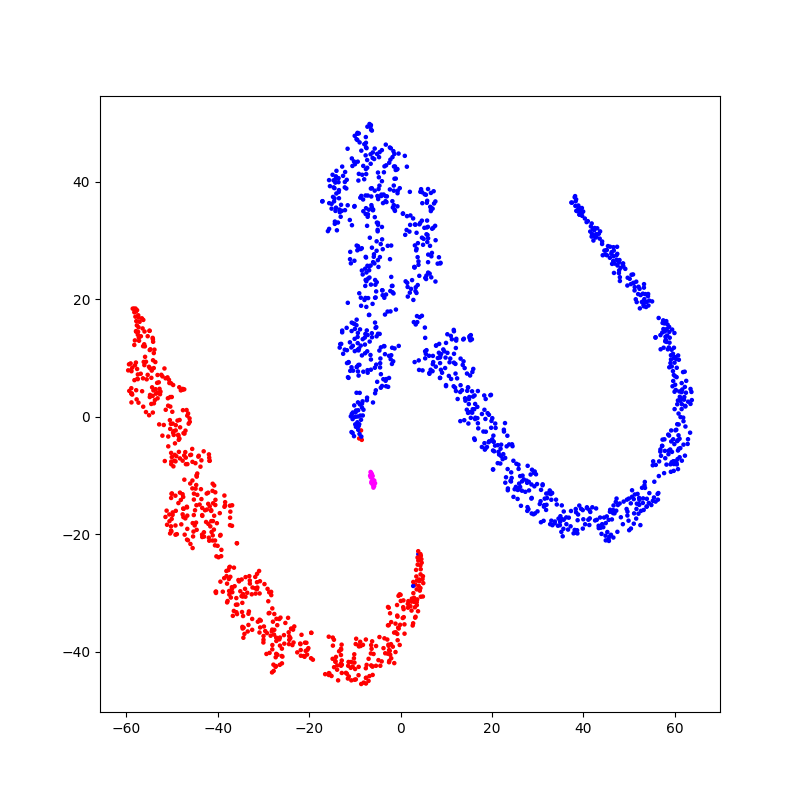 | Nzhl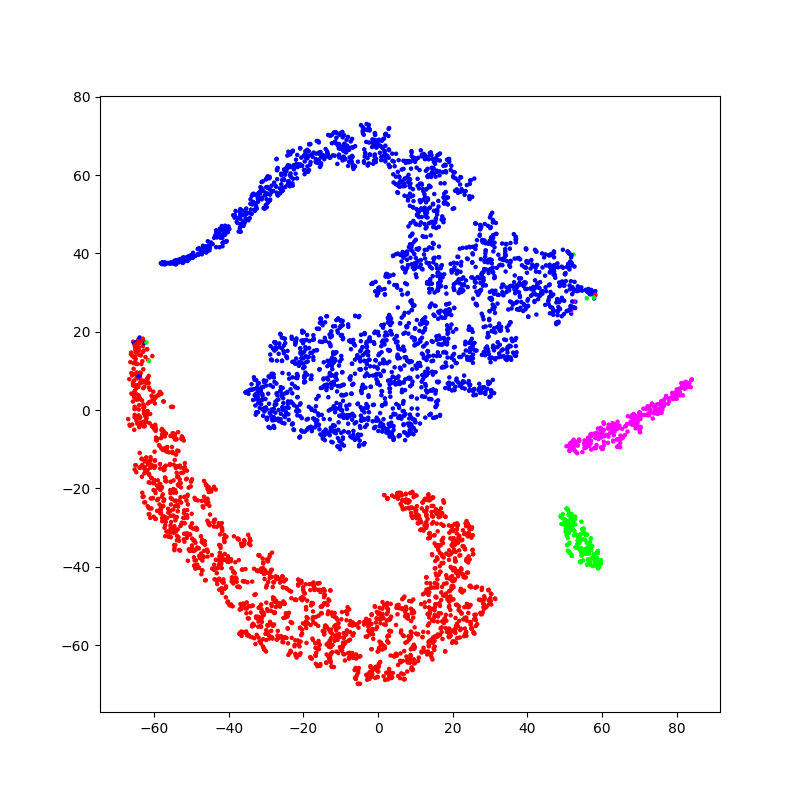 |
| RfL0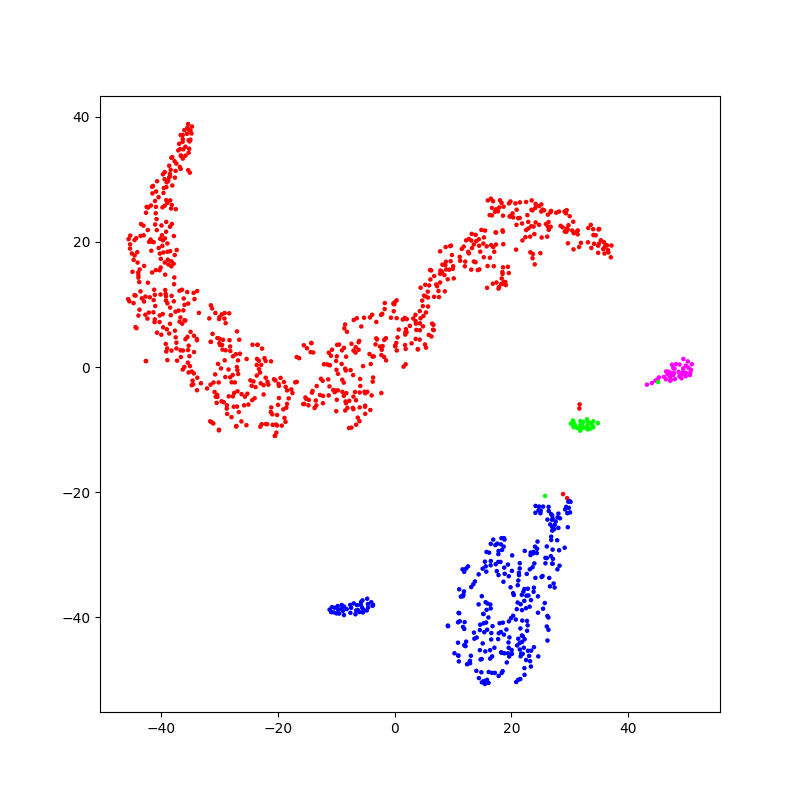 | svlu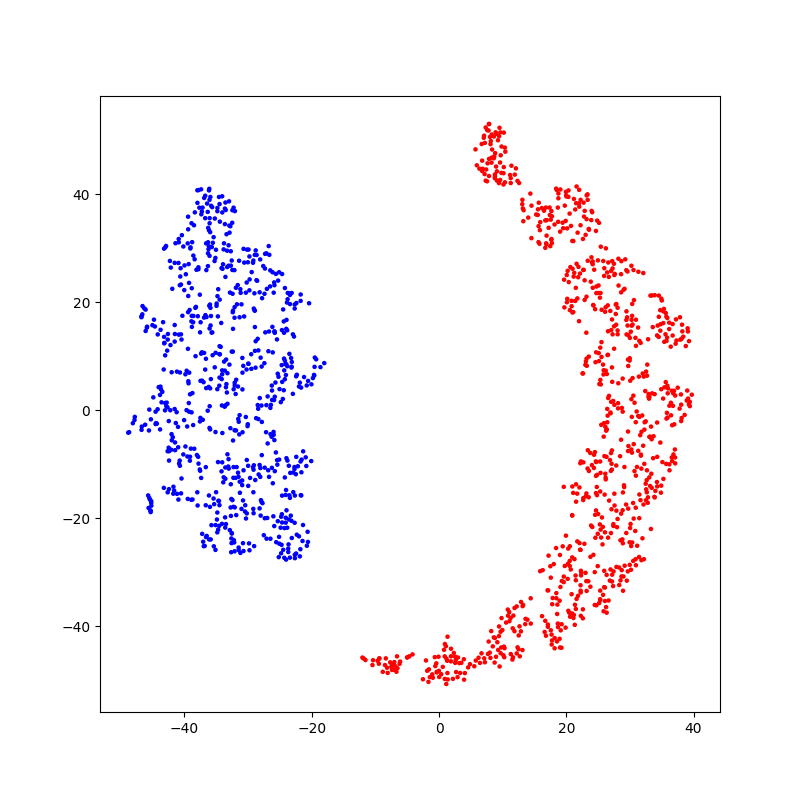 |
| Xg1l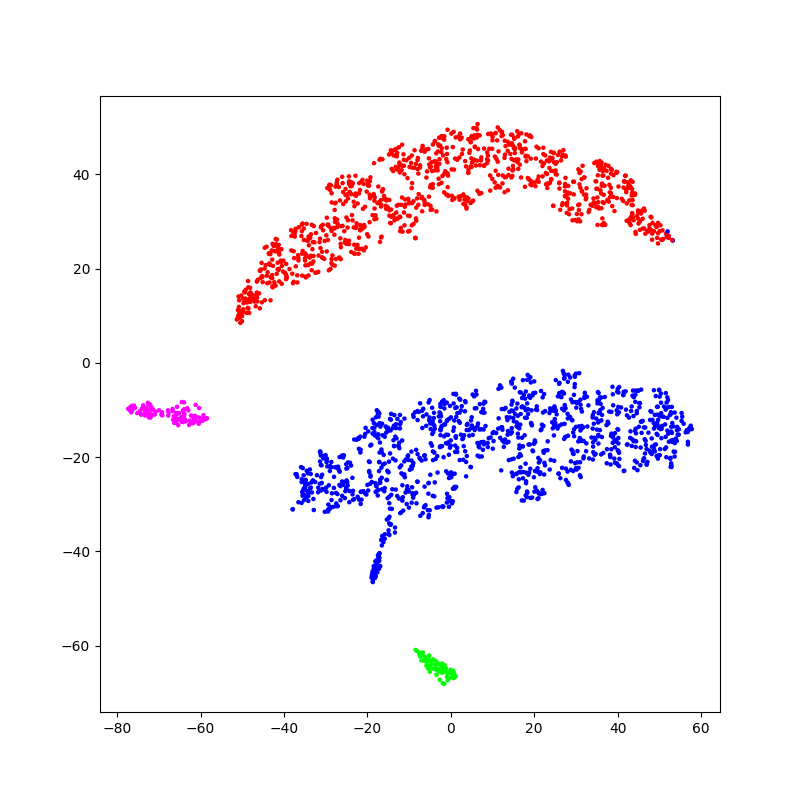 |  |

**Supplementary Figure S4.** T-distributed stochastic neighbor embedding (t-SNE) was used to illustrate the 12 patients of the validation dataset and their classification mapped into a 2D space (last layer of the CNN 16s; arbitrary units). Basically, two large clusters corresponding to W and MSE are visible which don’t completely separate. MSEc and ED don’t form clusters and are not separable from W and MSE. Wakefulness (W): blue; microsleep episodes (MSE): red; microsleep episode candidates (MSEc) green; episodes of drowsiness (ED): magenta. For the convenience we illustrated only every hundredth datapoint (sample). The patient ID is provided at the top of the plot. Please note that these figures only show the internal representation of the data in our specific network. Training data are illustrated in Supplementary Figure S3.

| ipxV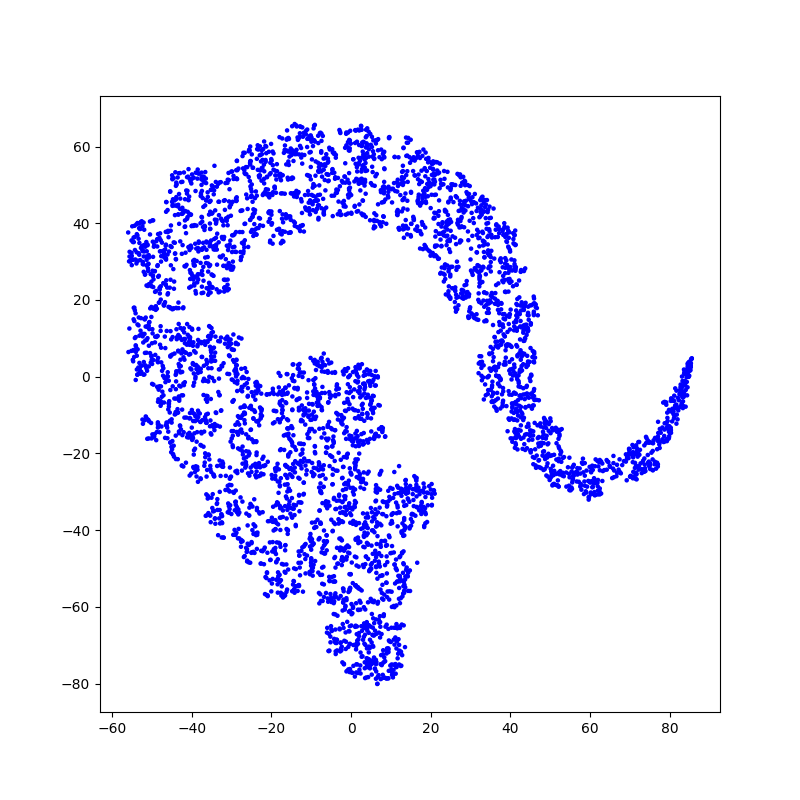 | RM1S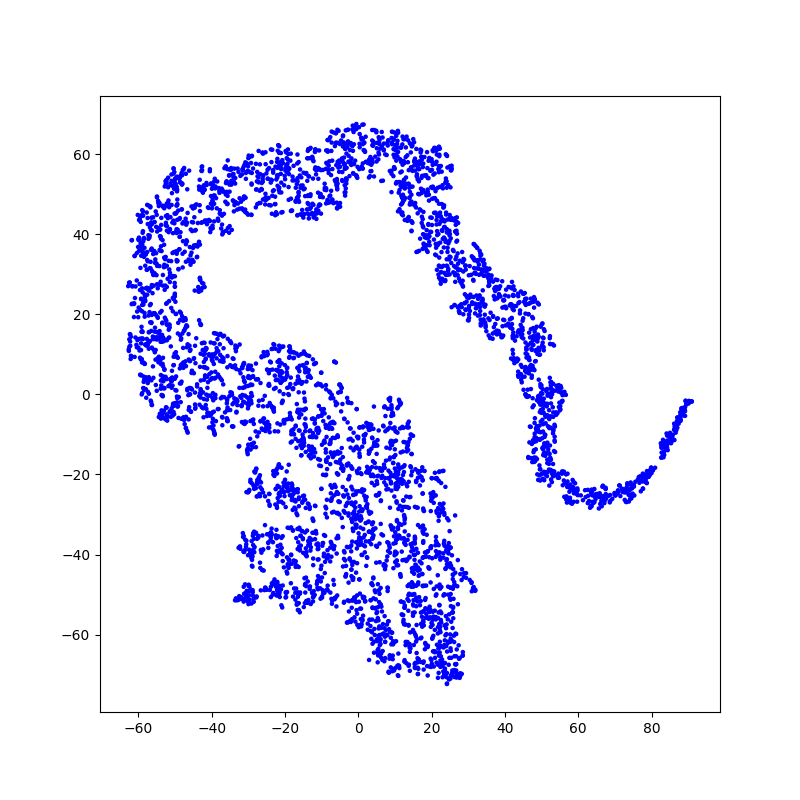 |
| --- | --- |
| AsLD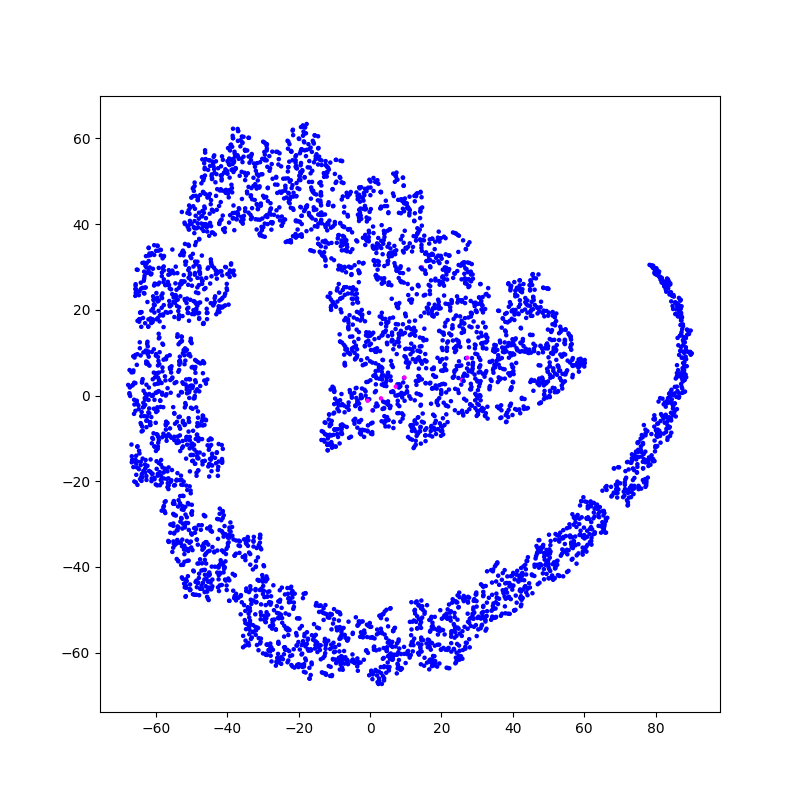 | d3ET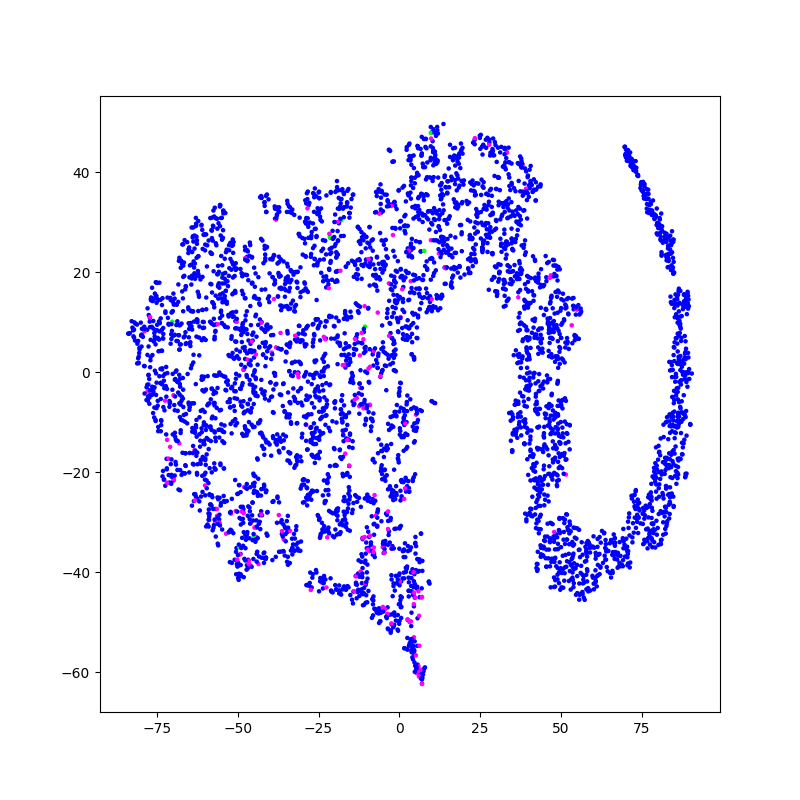 |

| uXdB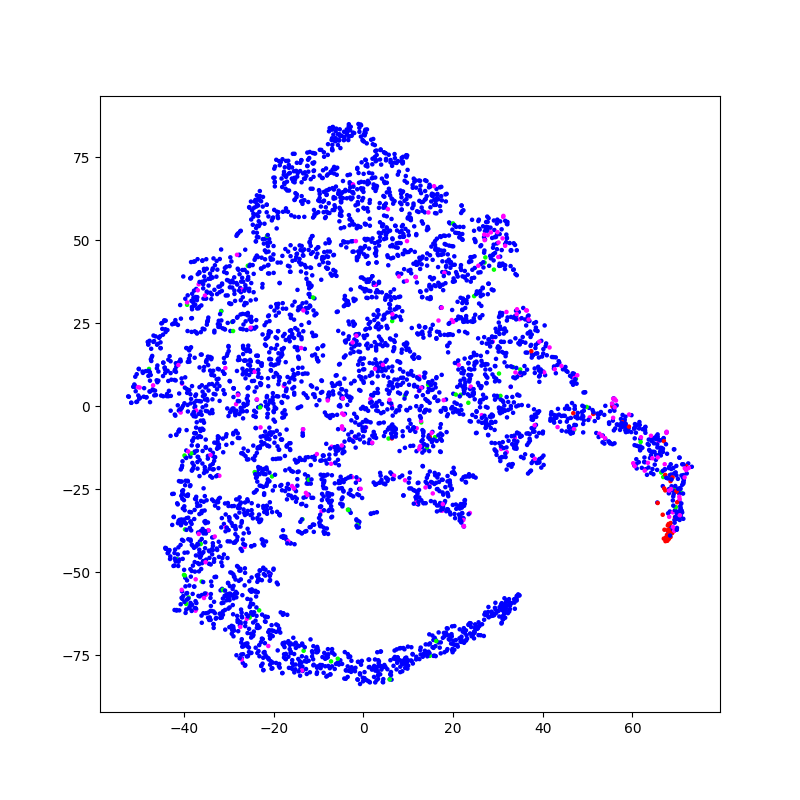 | 3J4W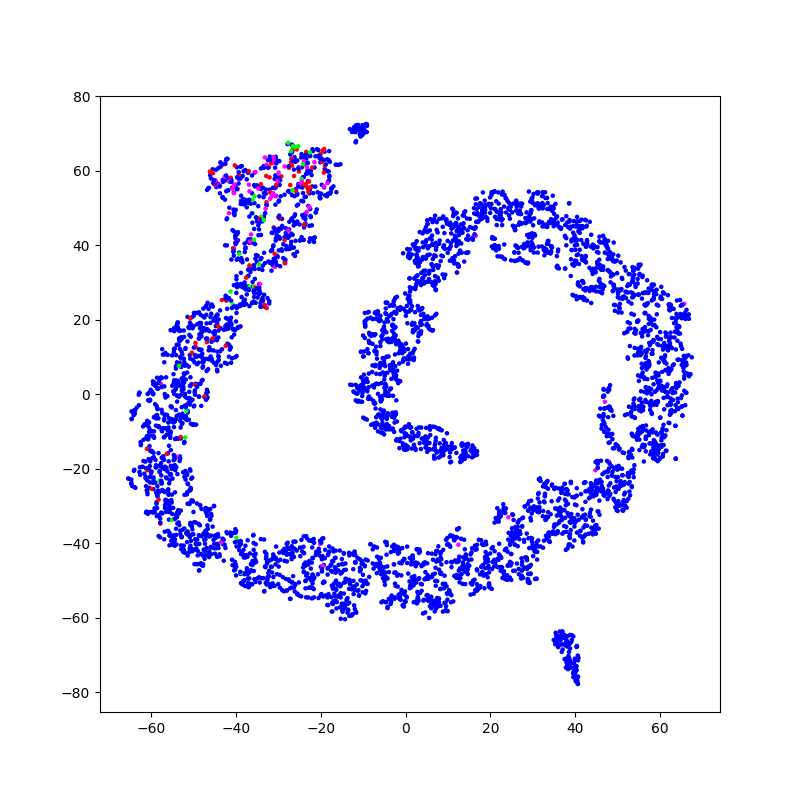 |
| --- | --- |
| zaca | 9JQY |
| 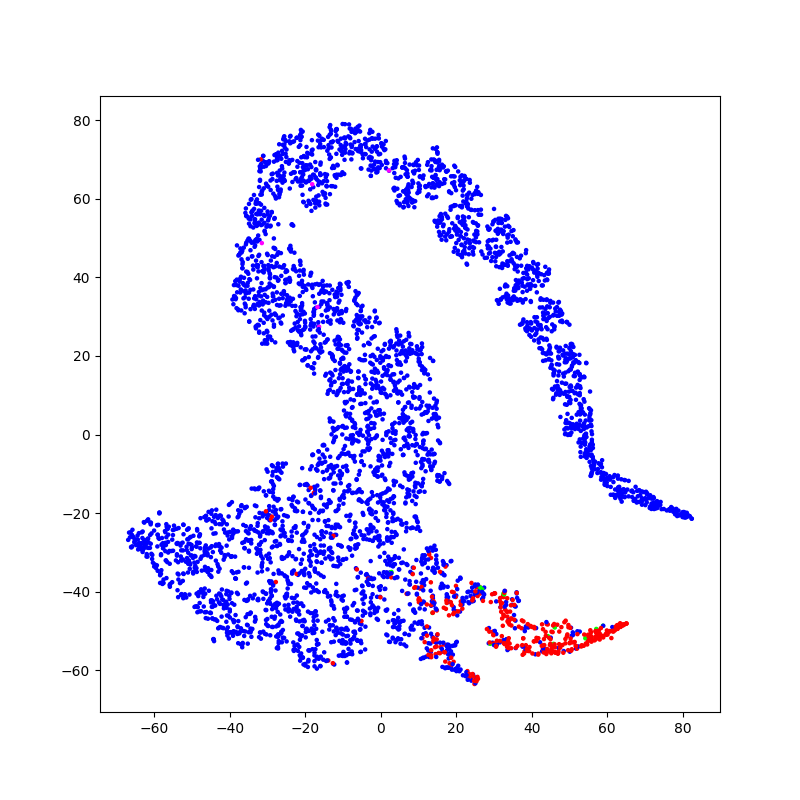 | 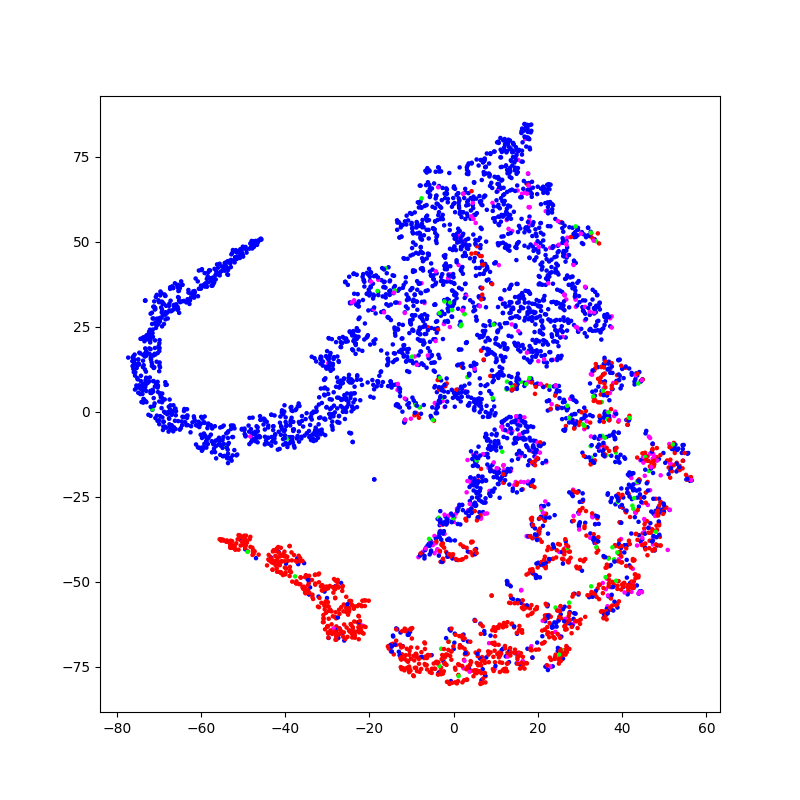 |

| JCpz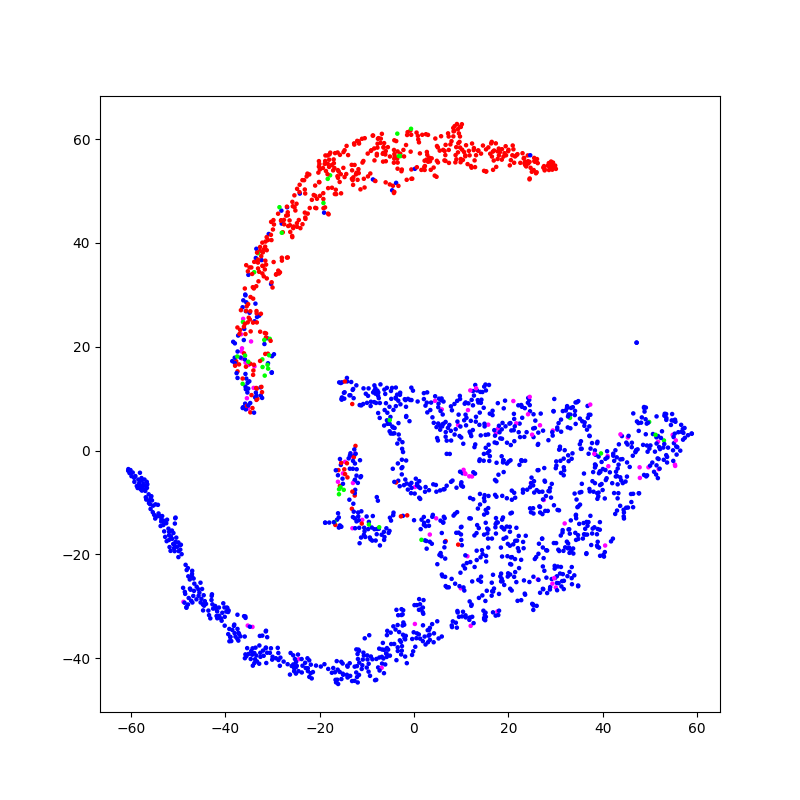 | oOMR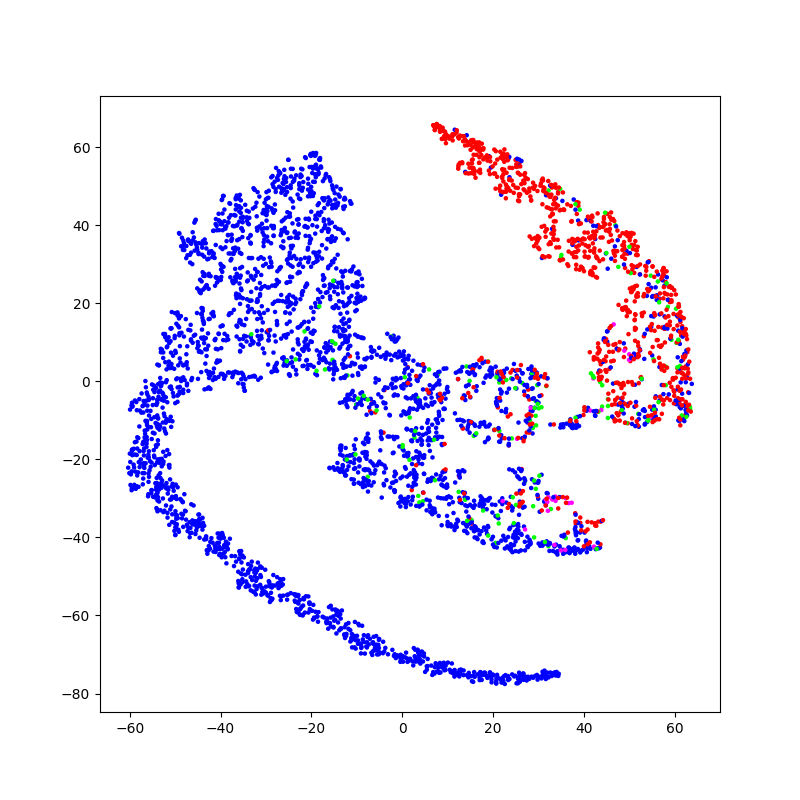 |
| --- | --- |
| sNMf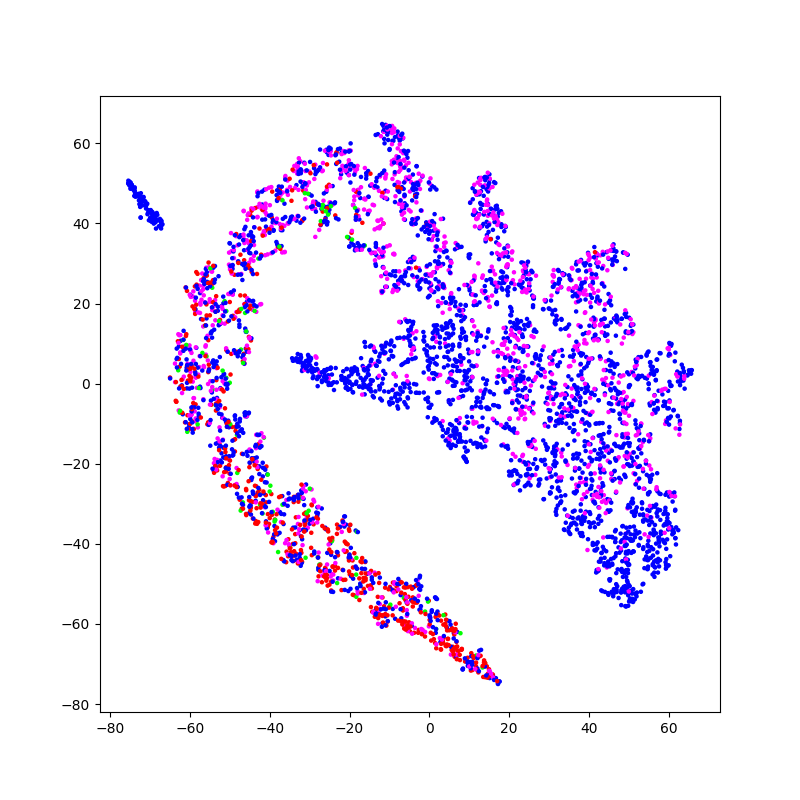 | Y5We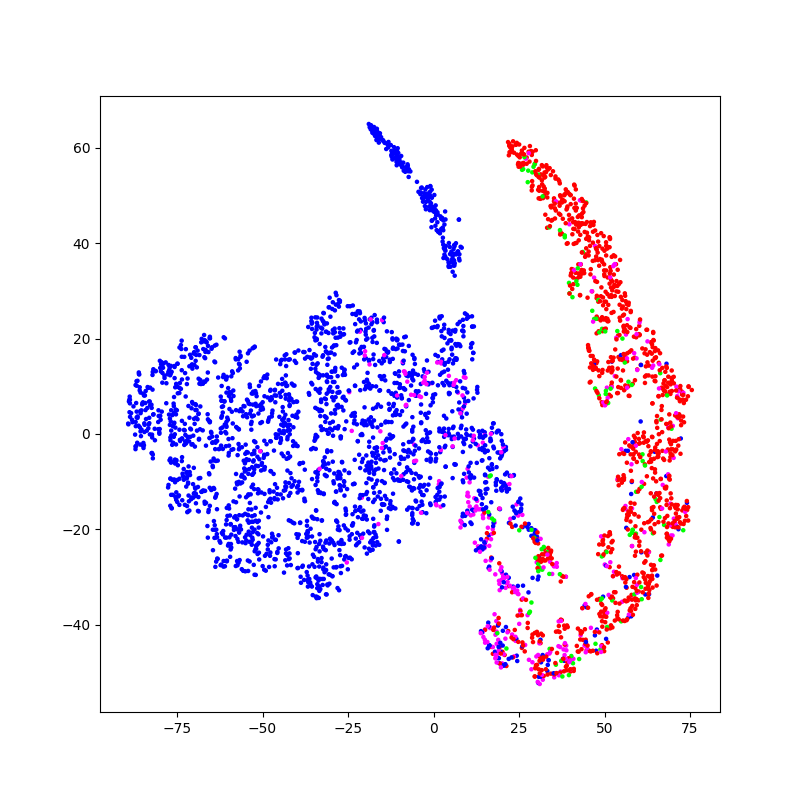 |
